# Mitochondrial Regulation of the NLRP3 Inflammasome in Diabetic Kidney Disease: From Mechanisms to Therapeutic Strategies

**DOI:** 10.3390/ijms27114819

**Published:** 2026-05-27

**Authors:** Xiangyu Chen, Zhenyu Wu, Kaiyan Yu, Juan Zhang, Hongjie Di

**Affiliations:** Department of Endocrinology, Jiangsu Province Second Hospital of Chinese Medicine (The Second Affiliated Hospital of Nanjing University of Chinese Medicine), Nanjing 210017, China; 18752402171@163.com (X.C.);

**Keywords:** NLRP3 inflammasome, diabetic kidney disease, mitochondria, pyroptosis, therapeutic strategies

## Abstract

Despite current therapies, persistent chronic inflammation remains an important contributor to residual renal risk in diabetic kidney disease (DKD). The NLRP3 inflammasome is a key driver of this inflammatory cascade, with mitochondria serving as a central hub that translates metabolic stress into NLRP3 activation via mitochondrial reactive oxygen species (mtROS) and oxidized mitochondrial DNA (ox-mtDNA). This review delineates the signaling network of the mitochondria–NLRP3 inflammasome axis in DKD and evaluates its potential as a therapeutic target to improve patient outcomes.

## 1. Introduction

Diabetic kidney disease (DKD) is a major microvascular complication of diabetes. Although current intervention strategies, including glycemic control, renin-angiotensin system (RAS) blockade, and emerging pharmacological agents such as sodium-glucose cotransporter 2 inhibitors and finerenone have substantially improved patient outcomes, a subset of patients remains at risk of disease progression and may ultimately develop kidney failure [1,2]. This residual renal risk suggests that additional pathological mechanisms driving DKD progression may not be fully addressed by existing therapeutic approaches [3]. Recent studies have indicated that persistent chronic inflammation within the kidney may be one of the key mechanisms underlying the sustained progression of DKD [3,4]. In this context, the NOD-like receptor family pyrin domain-containing 3 (NLRP3) inflammasome, an intracellular innate immune sensing platform, integrates a broad spectrum of danger signals, such as metabolic stress and oxidative injury, thereby promoting the maturation and release of interleukin-1β (IL-1β) and interleukin-18 (IL-18) and inducing pyroptosis [5]. Thus, the NLRP3 inflammasome is currently recognized as an important contributor to DKD progression.

How, then, do danger signals such as metabolic stress translate into sustained NLRP3 inflammasome activation? Converging evidence points to the mitochondria as a key answer to this question [6,7]. The kidney is an organ highly dependent on oxidative metabolism, rendering its mitochondria particularly vulnerable to metabolic insults such as hyperglycemia and lipid overload [8]. In DKD, damaged mitochondria constitute an upstream signaling network that promotes NLRP3 inflammasome activation through multiple pathways, including aberrant mitochondrial reactive oxygen species (mtROS) accumulation as well as the release of oxidized mitochondrial DNA (ox-mtDNA) and other mitochondria-derived damage-associated molecular patterns (DAMPs) [5]. Thus, mitochondria may function as an upstream organellar platform that couples diverse danger signals generated by metabolic injury to innate immune activation. Building upon this framework, this review takes the mitochondria as a central vantage point to overview the NLRP3 inflammasome and its roles in DKD, delineate the signaling network underlying the mitochondria–NLRP3 inflammasome axis, and explore the therapeutic status and prospects of targeting this axis.

## 2. Overview of the NLRP3 Inflammasome

The NLRP3 inflammasome is a multiprotein complex composed of three core components: the sensor protein NLRP3, the adaptor protein apoptosis-associated speck-like protein containing a CARD (ASC), and the effector precursor pro-caspase-1 [9]. NLRP3 expression and activation exhibit marked cell-type specificity, with NLRP3 being predominantly localized in the cytosol of myeloid immune cells, such as monocytes, macrophages, and dendritic cells of myeloid immune cells [10]. However, in the context of DKD, NLRP3 inflammasome can be induced and activated in resident renal cells, including podocytes, glomerular endothelial cells (GECs), mesangial cells (MCs), and tubular epithelial cells (TECs). These findings suggest that the NLRP3 inflammasome not only participates in the amplification of inflammation, but may also directly mediate renal structural and functional damage, thereby playing a pivotal role in DKD onset and progression [10,11].

**Figure 1:**
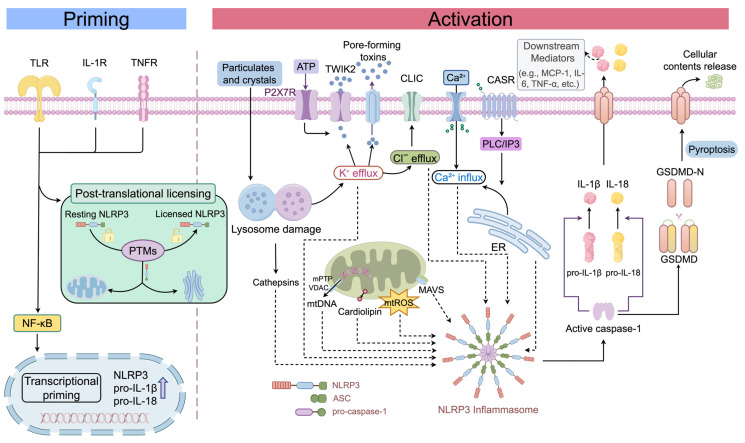
Canonical activation of the NLRP3 inflammasome. Canonical NLRP3 inflammasome activation involves two steps. Step 1 (Priming): TLR, IL-1R, or TNFR signaling via the NF-κB pathway drives the transcription of NLRP3 and pro-IL-1β/18. Concurrently, specific post-translational modifications (PTMs) regulate NLRP3 stability and protein interactions, while promoting its targeted recruitment to subcellular membrane compartments, including the TGN and mitochondria. Step 2 (activation): PAMPs or DAMPs trigger cellular stress signals (e.g., ionic imbalance, lysosomal rupture, mitochondrial dysfunction, and endoplasmic reticulum stress) to promote NLRP3 assembly with ASC and pro-caspase-1. Active caspase-1 then cleaves pro-IL-1β/18 and GSDMD, leading to cytokine release and pyroptosis. Created with Figdraw.

Over the past decade, the two-step model of NLRP3 inflammasome activation, comprising priming and activation, has been well established (Figure 1). The priming step is mainly mediated by Toll-like receptors (TLRs) and cytokine receptors, such as the tumor necrosis factor receptor (TNFR) and interleukin-1 receptor (IL-1R), which activate transcription factors including nuclear factor-κB (NF-κB). This leads to upregulation of NLRP3 and the precursor forms of its effector cytokines, pro-IL-1β and pro-IL-18, thereby establishing the transcriptional basis for subsequent inflammasome activation [9,12]. In addition to transcriptional induction, priming is accompanied by a subset of NLRP3 post-translational modifications (PTMs) associated with licensing. These modifications cooperatively regulate NLRP3 stability, subcellular localization, and interactions with binding partners, thereby conferring responsiveness to subsequent activating signals [13,14,15,16]. It should be noted that PTMs are not confined to the priming step. They also continue to regulate NLRP3 inflammasome assembly and activity during the activation step, thereby both preventing aberrant inflammasome activation and ensuring rapid assembly in the presence of activating stimuli [16]. During the activation step, pathogen-associated molecular patterns (PAMPs) or DAMPs elicit a range of molecular and cellular disturbances that culminate in NLRP3 inflammasome activation. These include disruption of ionic homeostasis—particularly K^+^ efflux, Ca^2+^ influx, and Cl^−^ efflux—along with mitochondrial dysfunction, lysosomal rupture, and endoplasmic reticulum stress [17,18]. Although the initiating stimuli are diverse, mitochondrial dysfunction occupies a central position across multiple pathological models [9,19,20,21].

During activation, NLRP3 undergoes a conformational rearrangement from an inactive double-ring cage-like structure into a disk-shaped active oligomer, thereby exposing its pyrin domain (PYD) [10]. This structural transition generally requires NIMA-related kinase 7 (NEK7) as a licensing factor [9]. Subsequently, NLRP3 recruits ASC via PYD–PYD interactions, promoting ASC oligomerization and CARD–CARD-mediated pro-caspase-1 recruitment, thereby driving inflammasome assembly and pro-caspase-1 autoactivation [10,18]. Activated caspase-1 cleaves pro-IL-1β and pro-IL-18 to generate their mature proinflammatory forms, while also cleaving gasdermin D (GSDMD) [9]. The liberated N-terminal fragment of GSDMD (GSDMD-NT) forms membrane pores that mediate the release of proinflammatory cytokines and induce pyroptosis [10].

## 3. NLRP3 Inflammasome in DKD

The NLRP3 inflammasome is not merely a marker of inflammatory responses in DKD but rather a key functional pathogenic factor driving disease onset and progression [5,22]. Earlier studies predominantly focused on its role in myeloid immune cells. However, accumulating evidence indicates that NLRP3 inflammasome activation in DKD is not confined to myeloid immune cells [11]. Studies using animal models and human kidney tissues have demonstrated that NLRP3 and its downstream effector molecules are expressed in multiple types of renal intrinsic cells, including podocytes [23,24,25], GECs [23,26,27], MCs [28,29], and TECs [24,30,31,32,33], and are closely associated with pathological alterations such as aggravated proteinuria, increased renal inflammation, tubulointerstitial fibrosis, and structural kidney damage. Notably, in *db/db* mice, the levels of NLRP3, IL-1β, IL-18, and related markers are already elevated prior to the onset of overt albuminuria and mesangial matrix expansion, suggesting that NLRP3 inflammasome activation may be involved in initiating the early pathological processes of DKD [23].

Furthermore, the pathological consequences of NLRP3 activation may differ across distinct cell types (Table 1) [11]. Current evidence suggests that NLRP3 activation in renal intrinsic cells may play a more direct pathogenic role in the development and progression of DKD. Bone marrow transplantation experiments have shown that, in *db/db* mice, selective deletion of *Nlrp3* or *Casp1* in bone marrow-derived cells alone does not significantly attenuate albuminuria or mesangial matrix expansion. Conversely, *Nlrp3*-deficient recipient mice that received wild-type bone marrow transplants still exhibited pronounced renoprotective effects [23]. These findings suggest that early glomerular injury in DKD may depend more critically on the NLRP3 axis within renal intrinsic cells. Future studies are warranted to further delineate key details, particularly the cell type-specific contributions and the dynamic changes that occur with disease stage progression, thereby providing more direct and actionable evidence for cell-stratified therapeutic strategies targeting NLRP3.

Overall, NLRP3 can be activated in both renal intrinsic cells and immune cells, contributing to DKD progression through multidimensional pathological networks. Based on this framework, the following section will systematically elaborate on the major roles of NLRP3 in DKD from the perspectives of pyroptosis, inflammatory responses, fibrosis, and crosstalk with other pathological mechanisms.

### 3.1. Cellular Pyroptosis in DKD

Pyroptosis, a form of inflammatory programmed cell death, has been increasingly recognized as a potential contributor to the onset and progression of DKD [11]. In the canonical pyroptosis pathway, the NLRP3 inflammasome promotes the activation of caspase-1, which subsequently cleaves GSDMD to generate the pore-forming GSDMD-NT. GSDMD-NT oligomerizes on the plasma membrane and forms transmembrane pores, leading to disruption of ionic homeostasis, cell swelling, and ultimately cell lysis [9]. Concurrently, pro-inflammatory cytokines and DAMPs are released into the extracellular space, further amplifying the local inflammatory response and potentially establishing a vicious cycle [11,32,55,56]. In addition, the non-canonical pyroptosis pathway—mediated by human caspase-4/5 or murine caspase-11 through GSDMD-dependent mechanisms—has also been implicated in podocyte injury associated with DKD, suggesting that distinct pyroptosis pathways may synergistically contribute to disease progression [57].

In the context of DKD, the pathological significance of pyroptosis should not be confined to the amplification of inflammatory signaling cascades; rather, emphasis should also be placed on its role as a lytic form of programmed cell death that directly depletes resident renal cells. This is particularly relevant for podocytes, which are terminally differentiated cells with limited proliferative and regenerative capacity; their sustained injury or loss is more prone to culminate in irreversible damage to the glomerular filtration barrier [58]. Multiple studies have demonstrated that both canonical and non-canonical GSDMD-dependent pyroptosis pathways are markedly activated in DKD animal models and in podocytes exposed to high-glucose conditions, accompanied by elevated levels of GSDMD-NT. These molecular alterations are closely associated with podocyte loss, foot process effacement, impairment of the filtration barrier, and exacerbation of proteinuria. Correspondingly, genetic knockout or silencing of the caspase-1/GSDMD signaling axis has been shown to partially attenuate these pathological changes [34,35,57]. Similarly, pyroptosis in TECs can result in brush border loss, disruption of tubular epithelial continuity, and structural damage such as tubular dilation and cast formation [46,47,48].

### 3.2. Maturation of Proinflammatory Cytokines and Amplification of Sterile Inflammation

During the progression of DKD, the renal inflammatory response is typically characterized by a chronic, low-grade, and sterile inflammatory state. In the absence of pathogenic infection, hyperglycemia, lipid accumulation, oxidative stress, tissue hypoxia, and various DAMPs can independently or synergistically provide the critical signals required for NLRP3 inflammasome activation [4,5]. Once activated, the NLRP3 inflammasome converts intracellular metabolic stress and danger signals into diffusible pro-inflammatory mediators by promoting the maturation and release of IL-1β and IL-18 as well as inducing pyroptosis, thereby amplifying the local inflammatory response [5,11].

Mature IL-1β primarily activates inflammatory transcriptional programs, including NF-κB, through the IL-1R1/MyD88-dependent signaling pathway, thereby inducing the expression of secondary pro-inflammatory mediators such as IL-6, tumor necrosis factor-α (TNF-α), and chemokines [59,60,61]. IL-18, in turn, activates NF-κB/activator protein-1 signaling via the IL-18R/MyD88 pathway and promotes interferon-γ-related inflammatory responses, collectively amplifying the inflammatory cascade in DKD [62]. These pro-inflammatory cytokines further upregulate the expression of adhesion molecules such as intercellular adhesion molecule-1 (ICAM-1) and chemokines, including monocyte chemoattractant protein-1 (MCP-1/CCL2) and C-X3-C motif chemokine ligand 1 (CX3CL1), thereby facilitating the recruitment, infiltration, and activation of immune cells—predominantly monocytes/macrophages—into the renal tissue [4,59,63]. Under sustained pro-inflammatory microenvironmental stimulation, infiltrating inflammatory cells and resident renal cells further amplify the production of chemokines and pro-inflammatory mediators, establishing a self-sustaining positive inflammatory feedback loop that perpetuates renal injury and drives the chronic inflammatory progression of DKD [4,63].

Furthermore, DAMPs released during pyroptosis, such as adenosine triphosphate (ATP), high mobility group box 1 (HMGB1), and mtDNA, can further stimulate neighboring cells [5,55,56,64]. Among these, HMGB1 primarily provides priming signals through TLRs or the receptor for advanced glycation end-products (RAGE), whereas extracellular ATP delivers the activation signal via the P2X7 receptor [56,64,65]. Collectively, these events establish conditions that favor a new round of NLRP3 inflammasome activation. This pyroptosis-mediated feed-forward circuit, involving DAMP release and inflammasome reactivation in neighboring cells, may help explain the persistence of inflammation in DKD under conditions of chronic metabolic stress.

### 3.3. NLRP3-Mediated Renal Fibrosis in DKD

As noted above, if the inflammatory response in DKD persists chronically or is repeatedly activated, it can establish a profibrotic microenvironment that drives the initiation and progression of renal fibrosis. During this process, injured or activated resident renal cells interact with infiltrating immune cells, continuously releasing pro-inflammatory cytokines, chemokines, and profibrotic mediators. These processes promote myofibroblast activation, disrupt extracellular matrix (ECM) homeostasis, and induce structural remodeling of renal tissue, ultimately leading to renal functional impairment [66,67]. NLRP3 plays a key regulatory role in the fibrotic process and may contribute to the regulation of renal fibrosis through both inflammasome-dependent and inflammasome-independent mechanisms [68].

Regarding the inflammasome-dependent pathway, beyond the overall effects of the inflammatory microenvironment, the core mechanism lies in the crosstalk between the effector molecules IL-1β and IL-18 and classical profibrotic signaling pathways such as transforming growth factor-β (TGF-β)/Smad [68]. Substantial evidence has shown that IL-1β can promote the phenotypic transition of renal tubular epithelial cells toward myofibroblasts, induce fibroblast proliferation, and enhance matrix protein synthesis [69,70]. Similarly, IL-18 has also been demonstrated to promote fibrotic phenotypic transition and collagen production in renal tubular epithelial cells [71]. In vivo experiments demonstrated that *Nlrp3* knockout significantly attenuated glomerulosclerosis, mesangial matrix expansion, and interstitial fibrosis in diabetic mice, while downregulating TGF-β1 expression and inhibiting Smad3 activation, thereby further confirming the pivotal role of NLRP3 in the progression of renal fibrosis in DKD [72].

In addition to the inflammasome-dependent pathways described above, NLRP3 itself may also directly regulate fibrosis-related signaling through inflammasome-independent mechanisms. Wang et al. found that, in TECs, NLRP3 upregulated TGF-β expression and promoted Smad2/3 phosphorylation, and that these effects were independent of caspase-1 activation and IL-1β/IL-18 processing [73]. Notably, this study was based on a unilateral ureteral obstruction model, and the applicability of its conclusions to DKD requires further validation. Moreover, NLRP3 and Smad2/3 have also been reported to exhibit intracellular spatial colocalization, further suggesting that NLRP3 may directly participate in the regulation of fibrotic signaling through inflammasome-independent pathways [68].

### 3.4. Crosstalk with Other Pathological Mechanisms

#### 3.4.1. Crosstalk Between the NLRP3 Inflammasome and Autophagy

The NLRP3 inflammasome and autophagy constitute a tightly interconnected bidirectional regulatory network [74]. Under physiological conditions, autophagy restrains aberrant NLRP3 activation by removing damaged mitochondria, reducing ROS burden, and promoting the degradation of inflammasome components such as ASC [75]. However, under hyperglycemic conditions, nutrient excess signals can activate the mechanistic target of rapamycin (mTOR) while suppressing nutrient- and energy-sensing pathways such as AMP-activated protein kinase (AMPK) and sirtuin 1 (Sirt1), thereby impairing autophagic flux [76]. Once autophagic function is compromised, the impaired clearance of damaged mitochondria and inflammasome components further potentiates NLRP3 inflammasome activation [74,77]. Relevant studies have shown that restoration of the autophagy-related CDKN1B/p70S6K signaling pathway may attenuate podocyte injury under high-glucose conditions and ameliorate DKD in STZ-treated mice by suppressing NLRP3 inflammasome activation [78].

Notably, excessive NLRP3 activation is not only a consequence of impaired autophagy but may also feed back to further suppress autophagic activity. It has been demonstrated that NLRP3 interacts with the autophagy-initiating protein Beclin-1 through its NACHT domain [79]. In DKD mouse models, silencing of the *Nlrp3* gene reversed the pathological alterations caused by its overactivation, improved autophagy-related indices such as the LC3-II/LC3-I ratio and Beclin-1 expression, promoted autophagosome formation, and alleviated podocyte injury [25]. In addition, activated caspase-1 may inhibit autophagy through cleavage of TRIF, whereas IL-1β may also modulate autophagic processes via the phosphatidylinositol 3-kinase (PI3K)/protein kinase B (AKT)/mTOR signaling pathway [75,80]. However, direct evidence for these mechanisms in DKD remains insufficient, and their precise causal relationships have yet to be fully elucidated.

#### 3.4.2. Crosstalk Between the NLRP3 Inflammasome and Lipid Dysregulation

Lipid metabolic dysregulation is a common pathological feature in patients with DKD. Ectopic lipid deposition in the kidney can induce lipotoxic renal injury, and the NLRP3 inflammasome may function as a critical molecular link between lipid dysregulation and renal injury [81]. First, multiple endogenous lipid species, including saturated fatty acids, oxidized low-density lipoprotein, and cholesterol crystals, can act as potent activators of the NLRP3 inflammasome, primarily through mechanisms involving stress responses in organelles such as mitochondria, the endoplasmic reticulum, and lysosomes [82]. In addition, certain proinflammatory adipokines, such as leptin and resistin, may facilitate the conversion of systemic metabolic disturbances into local renal inflammatory responses by activating intrarenal inflammatory signaling and promoting NLRP3 inflammasome activation [83,84,85]. Conversely, NLRP3 inflammasome activation may further aggravate lipid metabolic dysregulation. In DKD mouse models, inhibition or genetic deletion of NLRP3 markedly downregulated the expression of the lipogenic regulators sterol regulatory element-binding proteins 1 and 2 (SREBP1 and SREBP2), while upregulating the cholesterol transporter ATP-binding cassette transporter A1, thereby reducing glomerular cholesterol and triglyceride accumulation and ameliorating podocyte injury. Mechanistic studies indicate that this effect may involve the coordinated action of signaling networks centered on IL-1β, ROS, and NF-κB [36]. Similarly, Wang et al. reported that IL-1β increased the expression of the fatty acid transporter CD36 through activation of the NF-κB signaling pathway, thereby promoting the uptake of fatty acids and oxidized lipids and inducing lipotoxic injury in tubular cells [49].

#### 3.4.3. Crosstalk Between the NLRP3 Inflammasome and Oxidative Stress

Given that the following section will discuss in detail the role of ROS in activating the NLRP3 inflammasome, this subsection focuses on the pathological mechanisms by which NLRP3, in turn, exacerbates oxidative stress in the context of DKD. Both animal and cellular studies have confirmed the amplifying effect of NLRP3 on oxidative stress [36,72]. In mouse models of DKD and in vitro cell models, genetic deletion or pharmacological inhibition of NLRP3 significantly reduces intracellular ROS and superoxide levels, while also decreasing urinary excretion of the oxidative stress marker 8-hydroxydeoxyguanosine [36,72].

Mechanistically, Wu et al. approached this issue from the perspective of pro-oxidant enzymes and found that activation of the NLRP3 inflammasome may upregulate the expression of NADPH oxidase 4 (Nox4) through its downstream product IL-1β. Conversely, NLRP3 knockout or shRNA-mediated silencing effectively blocked high glucose-induced Nox4 expression and ROS burst [72]. Moreover, NLRP3 activation further impairs the endogenous antioxidant defense system of target cells. Subsequent studies in podocytes showed that targeted inhibition of the NLRP3 inflammasome effectively restored the expression of superoxide dismutases 1 and 2 (SOD1 and SOD2), which had been downregulated by high-glucose stimulation [36]. Notably, although thioredoxin-interacting protein (TXNIP) is recognized as a canonical upstream mediator linking ROS to NLRP3, recent evidence indicates that its expression is also subject to reciprocal regulation by NLRP3. Loss or silencing of *Nlrp3* markedly reciprocally suppresses TXNIP expression, suggesting the existence of a mutually reinforcing vicious cycle between NLRP3 and the ROS/TXNIP axis in DKD [72]. Taken together, NLRP3 is not merely a downstream sensor of ROS but also a critical driver that actively amplifies renal oxidative injury.

## 4. Mechanisms Underlying the Mitochondria–NLRP3 Inflammasome Axis in DKD

**Figure 2:**
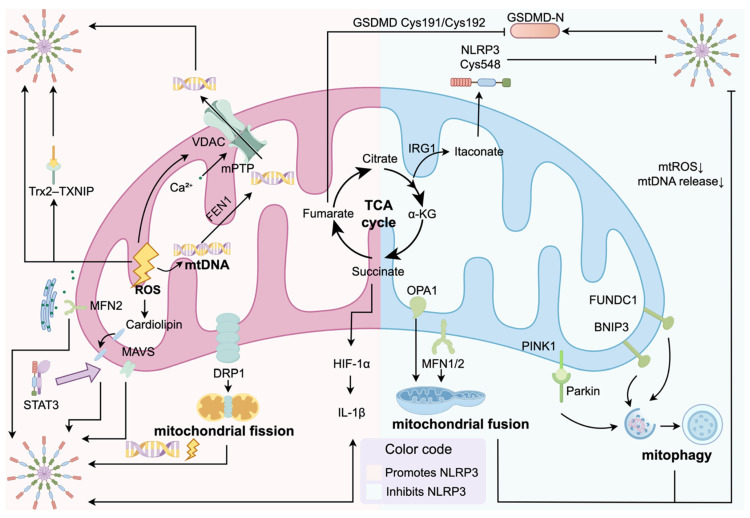
Proposed mechanistic model of mitochondrial regulation of NLRP3 inflammasome activation. Mitochondria regulate NLRP3 inflammasome activation through multiple interconnected mechanisms, including mitochondria-derived danger signals, mitochondrial dynamics, mitophagy, metabolic reprogramming, and mitochondria-associated signaling platforms. mtROS promotes TXNIP dissociation from the Trx–TXNIP complex and subsequent TXNIP binding to NLRP3, while direct oxidative regulation of NLRP3 remains a putative mechanism. Oxidized mtDNA can escape into the cytosol through mPTP/VDAC-dependent pathways, thereby acting as a mitochondria-derived danger signal. Pathological mitochondrial fission further disrupts mitochondrial membrane potential, increases mtROS generation, and facilitates the release of mtDNA and other mitochondrial DAMPs. Mitochondria-associated signaling platforms promote NLRP3 inflammasome assembly and activation: STAT3 facilitates NLRP3 translocation to mitochondria/MAMs; MAVS promotes NLRP3 recruitment to mitochondria; redistributed cardiolipin provides a lipid-binding platform for NLRP3 on damaged mitochondria; and MFN2 may facilitate NLRP3 inflammasome assembly/activation by associating with NLRP3, enhancing the NLRP3–MAVS interaction, and potentially participating in MAMs formation. Metabolic intermediates also participate in this regulation: succinate stabilizes HIF-1α and promotes IL-1β production potential, whereas fumarate and itaconate exert inhibitory effects by impairing GSDMD oligomerization/pore formation and disrupting the NLRP3–NEK7 interaction, respectively. Conversely, balanced mitochondrial fusion and mitophagy suppress sustained NLRP3 activation by maintaining mitochondrial network integrity, eliminating damaged mitochondria, reducing mtROS accumulation, and limiting the release of oxidized mtDNA and other mitochondria-derived DAMPs. Symbol legend: “→” indicates promotion; “⊣” indicates inhibition. Color legend: Pink mitochondria represent factors or conditions that promote NLRP3, whereas blue mitochondria represent factors or conditions that inhibit NLRP3. Created with Figdraw.

Mitochondria are not only central hubs of cellular energy metabolism but also key nodes for the integration and fine regulation of NLRP3 inflammasome signaling (Table 2; Figure 2). On the one hand, multiple danger signals released by damaged mitochondria can serve as direct triggers for NLRP3 activation [7]. On the other hand, the mitochondrial outer membrane and endoplasmic reticulum–mitochondria contact sites, also known as mitochondria-associated endoplasmic reticulum membranes (MAMs), can cooperate with organelles such as the trans-Golgi network (TGN) to provide physical scaffolds for the subcellular localization and assembly of NLRP3, thereby contributing to the spatiotemporal regulation of its activation [86]. Under physiological conditions, quality-control systems such as mitophagy limit aberrant amplification of inflammation by eliminating damaged mitochondria [87].

**Figure 3:**
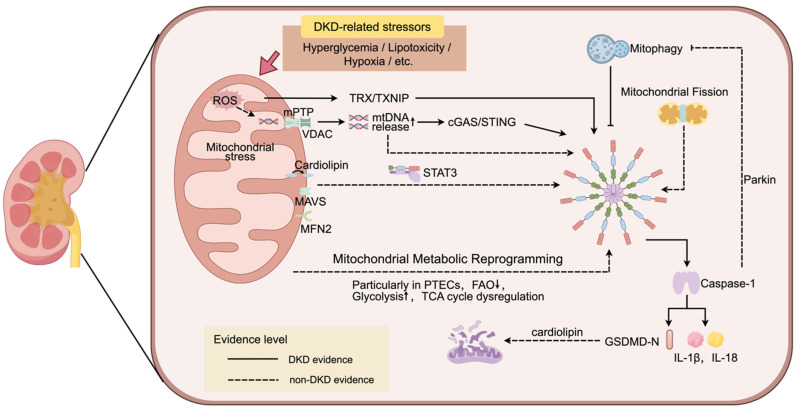
Proposed integrated model of the mitochondria–NLRP3 inflammasome axis in DKD. M1: mtROS and mtDNA have been shown to contribute to NLRP3 activation in DKD. mtROS promotes TXNIP dissociation from the Trx–TXNIP complex, enabling TXNIP–NLRP3 interaction. mtDNA activates the inflammasome via the cGAS–STING pathway and may also directly trigger NLRP3 inflammasome activation under certain conditions. M2: Mitochondria and MAMs as spatial platforms for NLRP3 inflammasome assembly. Upon activating stimulation, NLRP3 is recruited to the outer mitochondrial membrane to facilitate full macromolecular assembly. STAT3 phosphorylation at Ser727 enhances the STAT3–NLRP3 interaction and promotes mitochondrial translocation of NLRP3. In parallel, cardiolipin, MAVS, and MFN2 collectively facilitate NLRP3 localization and inflammasome assembly. M3: In DKD, metabolic stress induces DRP1-dependent mitochondrial fission and mitochondrial fragmentation, resulting in mitochondrial dysfunction, mtROS overproduction, membrane permeabilization, and mtDNA release, which promote sustained NLRP3 inflammasome activation and subsequent renal inflammation, fibrosis, and injury. In contrast, PINK1/Parkin-mediated mitophagy removes damaged mitochondria, reduces mtROS and mitochondrial DAMP release, and suppresses NLRP3 activation. M4: In PTECs, high glucose drives metabolic reprogramming marked by impaired FAO, enhanced glycolysis, TCA cycle dysfunction, and lipid accumulation, which contribute to NLRP3 inflammasome activation and inflammatory tubular injury. M5: During pyroptosis, activated GSDMD-NT binds cardiolipin and forms pores in mitochondrial membranes, leading to mitochondrial damage, ROS accumulation, membrane potential loss, impaired OXPHOS, and release of mitochondrial proteins and mtDNA. Caspase-1 may also inactivate Parkin, reducing the clearance of damaged mitochondria and further amplifying inflammation. Symbol legend: “→” indicates promotion; “⊣” indicates inhibition. Dashed lines indicate mechanisms supported mainly by non-renal or non-DKD studies. Created with Figdraw.

However, under the chronic metabolic stress characteristic of DKD, the mitochondria–NLRP3 inflammasome axis is highly prone to pathological dysregulation. Because the kidney relies heavily on oxidative phosphorylation for energy production, it is particularly sensitive to disturbances in mitochondrial homeostasis [8]. Mitochondria are also considered key organelles that are affected early in DKD and participate in disease initiation and progression [120]. In DKD-related metabolic stress environments, such as persistent hyperglycemia and lipotoxicity, intrinsic renal cells are chronically exposed to an overload of glucose, fatty acids, and their metabolic intermediates, resulting in a marked increase in mitochondrial metabolic burden [8]. Continuous influx of excess substrates into mitochondria places the electron transport chain (ETC) under high load, thereby promoting electron leakage and mtROS generation [120,121]. Meanwhile, sustained metabolic stress may also be accompanied by metabolic reprogramming and impaired mitochondrial quality control, which manifests as reduced oxidative phosphorylation efficiency, excessive mitochondrial fission, and defective mitophagy [8,120]. In this microenvironment, mitochondria undergo a functional transition from victims of injury to drivers of inflammation: damaged mitochondria that cannot be cleared promptly release abundant danger signals, such as excessive mtROS and leaked ox-mtDNA, thereby robustly promoting NLRP3 inflammasome activation [7]. Notably, downstream effector molecules generated after NLRP3 inflammasome activation can, in turn, further damage mitochondria, thereby reinforcing the positive feedback loop between mitochondrial injury and inflammatory activation [122,123]. This section focuses on the potential mechanisms by which mitochondrial signals drive NLRP3 inflammasome activation and, conversely, how NLRP3 activation disrupts mitochondrial homeostasis in the context of DKD (Figure 3).

### 4.1. Mitochondrial Danger Signals in NLRP3 Regulation

#### 4.1.1. mtROS

In the context of this metabolic vulnerability, mtROS emerge early as functionally important mitochondrial danger signals linking mitochondrial stress to NLRP3 inflammasome activation in DKD [5,8]. Persistent hyperglycemia, lipotoxicity, and hypoxic or pseudohypoxic stress can disrupt mitochondrial redox homeostasis and electron transport chain function, thereby favoring mitochondrial membrane potential disturbance, electron leakage, and ROS generation, particularly involving complexes I and III [121]. Meanwhile, impaired fatty acid oxidation, defective oxidative phosphorylation, weakened mitochondrial antioxidant systems, and altered SIRT3-dependent protein deacetylation further aggravate mitochondrial redox imbalance, collectively driving mtROS accumulation in DKD [8,121,124,125].

Accumulating evidence indicates that mtROS contribute to NLRP3 inflammasome activation at multiple steps. During the priming stage, ROS can enhance NF-κB-dependent transcriptional priming, thereby promoting the expression of key components such as NLRP3 and pro-IL-1β [126,127]. In addition to transcriptional priming, ROS have been implicated in the non-transcriptional licensing of NLRP3 in certain experimental settings, including deubiquitination-related events and ROS–MINK1 axis-dependent phosphorylation of NLRP3 at Ser725, although the relevance of these mechanisms in DKD requires further clarification [128,129]. During the activation stage, the TRX/TXNIP axis has been proposed as an important molecular link between mtROS generation and NLRP3 inflammasome activation. Under oxidative stress conditions, TXNIP dissociates from thioredoxin (TRX) and interacts with NLRP3, thereby promoting inflammasome assembly, caspase-1 activation, and the maturation and release of IL-1β and IL-18 [21]. Excessive mtROS may also oxidize mitochondrial DNA and promote oxidized mtDNA release, further amplifying NLRP3 activation [7]. In DKD-related animal and cell models, sustained activation of the TXNIP–NLRP3–IL-1β axis has been observed, and antioxidant intervention can attenuate this response, supporting the pathogenic relevance of the mtROS–TXNIP–NLRP3 pathway [41,130,131].

Based on these mechanisms, targeting mtROS and restoring mitochondrial redox homeostasis may have therapeutic potential in DKD. Mitochondria-targeted antioxidants, such as MitoTEMPO, have been shown to suppress mtROS-related NLRP3 activation and reduce albuminuria in preclinical DKD models [23,48]. Enhancement of endogenous antioxidant defenses, such as Nuclear factor erythroid 2-related factor 2 (Nrf2)-mediated antioxidant signaling, may also improve redox homeostasis and maintain mitochondrial integrity, thereby potentially exerting inhibitory regulation on NLRP3 inflammasome activation in DKD by reducing mtROS accumulation and TXNIP expression [120,132,133,134]. In addition, modulation of mitochondrial protein acetylation represents a complementary strategy. Restoration of sirtuin 3 (SIRT3), a major mitochondrial NAD^+^-dependent deacetylase, may improve mitochondrial antioxidant capacity by deacetylating antioxidant proteins such as SOD2 and by preserving oxidative phosphorylation efficiency, thereby reducing mtROS generation and secondarily limiting NLRP3 inflammasome activation [125,135]. Taken together, mtROS are not merely toxic byproducts of metabolic stress, but rather the master regulators orchestrating NLRP3 inflammasome hyperactivation, thereby fueling the progression of diabetic renal injury.

#### 4.1.2. mtDNA

Under oxidative stress, mitochondrial damage is progressively exacerbated and accompanied by abnormal increases in mitochondrial membrane permeability, leading to the cytosolic release of mtDNA, particularly ox-mtDNA. Once in the cytosol, mtDNA serves as a prototypical DAMP that initiates and amplifies inflammatory responses through multiple signaling pathways, including the NLRP3 inflammasome [88,136]. Mechanistically, opening of the mitochondrial permeability transition pore (mPTP) and oligomerization of the voltage-dependent anion channel (VDAC) may together provide the structural basis for the translocation of ox-mtDNA fragments across mitochondrial membranes into the cytosol [91,92]. In addition, by manipulating multiple DNA-processing factors, Xian et al. found that flap endonuclease 1 (FEN1) can process mtDNA into fragments of approximately 500–650 bp, thereby rendering them more compatible with the size requirements for transmembrane transport and cytosolic release [91]. These findings further suggest that alterations in mitochondrial membrane permeability and mtDNA release should not be viewed merely as passive consequences of end-stage cellular injury, but rather as pivotal events linking oxidative stress, mitochondrial damage, and inflammasome activation.

Within the setting of DKD, the cGAS/STING pathway may serve as a key regulatory hub linking cytosolic mtDNA signaling to NLRP3 inflammasome activation [137]. Available studies suggest that cGAS/STING signaling is upregulated in DKD-related cellular and animal models and exhibits changes associated with disease progression; moreover, STING deficiency is accompanied by reduced NLRP3 inflammasome activation and can alleviate renal inflammation as well as structural and functional injury [138,139]. Evidence for crosstalk between cGAS/STING signaling and the NLRP3 inflammasome is also steadily accumulating [136,140]. Other studies further suggest that, in certain sterile inflammatory models, oxidized mtDNA may participate in NLRP3 inflammasome activation in a cGAS-independent manner [136]. This indicates that, although cGAS/STING may represent an important upstream pathway, it is unlikely to be the only one involved. Therefore, based on the currently available fragmentary evidence, a mechanistic framework can be proposed that warrants further validation: following its release into the cytosol, mtDNA—particularly ox-mtDNA—may promote the amplification of renal inflammation in DKD either through crosstalk between cGAS/STING and NLRP3 or directly via the NLRP3 inflammasome pathway. However, the specific mechanisms through which these two potential pathways operate in DKD, as well as their causal relationships, have not yet been directly confirmed experimentally.

### 4.2. Potential Roles of Mitochondria in NLRP3 Localization and Assembly in DKD

As discussed above, mitochondria-derived danger signals, such as mtROS and mtDNA, are important triggers of NLRP3 inflammasome activation in DKD [5]. However, how these signals are translated into the efficient assembly of NLRP3, ASC, caspase-1, and other inflammasome components remains dependent on more refined spatial regulatory mechanisms. Recent studies suggest that mitochondria and MAMs may serve as subcellular platforms for NLRP3 localization, recruitment, and inflammasome assembly [86]. Experimental studies in DKD and other kidney disease models have observed NLRP3 enrichment in mitochondrial regions or enhanced colocalization with mitochondrial markers, providing preliminary support for the pathological relevance of this spatial localization mechanism in renal inflammatory injury [141,142,143]. Nevertheless, whether these observations represent functional assembly sites and whether they depend on MAMs, mitochondrial antiviral-signaling protein (MAVS), cardiolipin, signal transducer and activator of transcription 3 (STAT3), or related mechanisms remains to be further validated.

At present, most DKD studies remain limited to colocalization observations or the assessment of inflammasome activity after experimental intervention. Systematic evidence is still lacking regarding the temporal dynamics, functional necessity, and molecular anchoring mechanisms of NLRP3 mitochondrial recruitment. Therefore, the mechanistic interpretation of how mitochondria/MAMs regulate NLRP3 spatial localization still needs to be informed by evidence from canonical inflammasome models. Further mechanistic studies suggest that mitochondrial localization of NLRP3 is not a static event, but may represent part of a sequential organelle translocation process during inflammasome assembly. A live-cell multichannel time-lapse imaging study showed that, under specific stimulatory conditions, NLRP3 first translocates to mitochondria approximately 10–15 min after stimulation and is subsequently recruited to the dispersed trans-Golgi network (dTGN) [144]. This finding suggests that NLRP3 inflammasome assembly does not occur at a single fixed site, but instead involves dynamic trafficking and coordination among mitochondria, Golgi-related membrane structures, and other organelle platforms. In the context of DKD, this mechanism provides a useful reference for understanding the observed mitochondrial enrichment of NLRP3. However, its temporal sequence and functional necessity still require direct validation in DKD-relevant renal cells.

With regard to molecular anchoring mechanisms, factors such as STAT3, cardiolipin, MAVS, and mitofusin 2 (MFN2) may participate in the recruitment and stabilization of NLRP3 at mitochondria [86]. Among them, cardiolipin, MAVS, and MFN2 have been shown to act as mitochondria-associated anchoring platforms that interact with NLRP3 and thereby promote its localization to mitochondrial regions [117,118,119]. Recent studies have further revealed a regulatory mechanism underlying NLRP3 mitochondrial translocation: upon NLRP3-activating stimulation, STAT3 undergoes phosphorylation at Ser727, which enhances its interaction with NLRP3 and subsequently promotes NLRP3 translocation to mitochondria; blockade of the STAT3–NLRP3 interaction attenuates downstream inflammatory effector responses [116]. Together, these findings indicate that mitochondrial localization of NLRP3 may depend on specific lipid microenvironments and protein interaction networks, rather than being passively driven solely by mitochondrial damage signals. These studies provide an important spatial biology framework for understanding the coupling between mitochondrial injury and NLRP3 inflammasome assembly, but this framework still requires further validation in DKD models. Future studies should systematically evaluate the dynamic recruitment of NLRP3 to mitochondria/MAMs under DKD-related stimuli and clarify whether this spatial translocation influences downstream events such as ASC speck formation, caspase-1 activation, IL-1β/IL-18 maturation, and DKD progression.

### 4.3. Mitochondrial Quality Control in NLRP3 Regulation

Mitochondrial quality control is an integrated network composed of mitochondrial dynamics, mitophagy, mitochondrial biogenesis, and other homeostatic maintenance mechanisms [121]. In DKD, this network is particularly important for the regulation of the NLRP3 inflammasome. Its central role is not merely to exert a general anti-inflammatory effect, but rather to limit the accumulation of damaged mitochondria, reduce mtROS production, and suppress the cytosolic release of mtDNA and other mitochondria-derived DAMPs, thereby attenuating the priming, assembly, and sustained activation of the NLRP3 inflammasome [87,145,146,147].

In DKD, multiple metabolic stressors can disrupt the balance of mitochondrial dynamics, as reflected by enhanced dynamin-related protein 1 (DRP1)-dependent excessive mitochondrial fission, together with reduced expression or impaired function of mitochondrial fusion-related proteins such as MFN1/2 and optic atrophy 1 (OPA1) [8]. Such dysregulation of mitochondrial dynamics leads to fragmentation of the mitochondrial network, decreases mitochondrial membrane potential, and compromises membrane integrity, thereby amplifying mitochondria-derived upstream activating signals for NLRP3 [98,99]. Studies in DKD-related models have demonstrated that interventions targeting DRP1-mediated aberrant mitochondrial fission can suppress NLRP3 inflammasome activation and its downstream inflammatory effects, while improving renal tissue injury and fibrotic phenotypes [145]. It should also be noted that, although enhanced fission and insufficient fusion are more commonly observed in DKD, from the perspective of mitochondrial quality control, unidirectional inhibition of fission or promotion of fusion is not necessarily beneficial. Although relevant evidence remains limited, existing studies suggest that mitochondrial fusion beyond physiological limits may also impair mitochondrial function and participate in the pathogenesis of DKD [148]. Therefore, interventions targeting mitochondrial dynamics in DKD should be understood primarily as strategies to restore the dynamic balance between fission and fusion, thereby reducing mitochondria-derived danger signals required for the sustained activation of the NLRP3 inflammasome.

At the level of mitochondrial biogenesis, peroxisome proliferator-activated receptor γ coactivator-1α (PGC-1α) is a key regulatory factor [121]. Impaired PGC-1α function may lead to insufficient mitochondrial renewal and an increased burden of damaged mitochondria, thereby enhancing mitochondria-derived activating signals for the NLRP3 inflammasome. Conversely, restoration of PGC-1α-related signaling may activate mitochondrial biogenesis programs, correct mitochondrial dynamics imbalance, preserve mitochondrial structural and functional integrity, and reduce mtROS production and mtDNA release into the cytosol. Through these mechanisms, PGC-1α signaling may suppress NLRP3-associated inflammatory and profibrotic effects and improve renal injury [149]. However, it should be noted that this evidence is not primarily derived from DKD models; therefore, its applicability to DKD remains to be further validated.

Similarly, mitophagy, particularly the PTEN-induced putative kinase 1 (PINK1)/Parkin axis, serves as an important negative regulatory mechanism that limits the persistent amplification of inflammasome signaling. By recognizing and selectively eliminating damaged mitochondria, this process reduces mtROS accumulation and restricts the release of mtDNA and other DAMPs, thereby limiting sustained NLRP3 activation at its source [7]. Analyses of renal biopsy samples have shown that Parkin expression is downregulated in patients with DKD, and lower Parkin levels are associated with more severe inflammatory/fibrotic phenotypes and poorer renal functional parameters [150]. In animal and cellular models of DKD, multiple interventions have been reported to restore or enhance PINK1/Parkin-mediated mitophagy, accompanied by reductions in inflammasome-related markers and improvements in renal injury phenotypes [146,147]. These findings provide further support for the view that enhancing mitochondrial quality control may help attenuate NLRP3 inflammasome-driven pathological processes in DKD at their source.

### 4.4. Mitochondrial Metabolic Reprogramming and NLRP3 Inflammasome

The kidney is a highly energy-demanding organ, with proximal tubules being particularly dependent on mitochondrial oxidative metabolism and fatty acid oxidation (FAO) for energy supply [8]. Under chronic stress conditions such as hyperglycemia and hypoxia, some renal resident cells may exhibit suppressed oxidative phosphorylation (OXPHOS) accompanied by enhanced glycolysis. Although this shift may be compensatory in the short term, its persistence is often associated with cellular injury and pathological remodeling [151]. Analysis of renal biopsy specimens from young adults with type 1 diabetes mellitus further showed that, even before the onset of clinical albuminuria, cortical oxidative metabolism had already begun to decline, accompanied by downregulation of transcriptional signatures related to the tricarboxylic acid (TCA) cycle and OXPHOS in proximal tubules [152]. Available evidence indicates that, in a variety of inflammatory models, enhanced glycolysis is commonly accompanied by activation of the NLRP3 inflammasome. However, given that certain glycolytic enzymes possess complex physiological functions beyond their canonical metabolic catalytic roles, interventions directed at different metabolic nodes may lead to heterogeneous outcomes across cell types and in different stimulus settings [151,153,154]. In addition, multi-omics and spatial metabolomics analyses have shown that proximal tubular epithelial cells (PTECs) in human kidney disease samples exhibit clear impairment of FAO, accompanied by accumulation of lipids and related metabolic intermediates, which may further aggravate tubular cell injury [155]. Hou et al. reported that both *db/db* mice and HK-2 cells cultured under high-glucose conditions exhibited a shift in metabolic substrate preference from FAO to glycolysis, accompanied by increased mtROS production and enhanced NLRP3/IL-1β signaling; notably, CD36 inhibition enhanced AMPK activity, partially restored mitochondrial FAO, and mitigated these inflammatory responses [48]. This study provides important experimental support for a link between FAO dysregulation and NLRP3 activation in the context of DKD. Although studies specifically addressing this issue in DKD remain relatively limited, available evidence suggests that maintenance of cellular metabolic and energetic homeostasis may help restrain excessive NLRP3 activation.

Meanwhile, studies using inflammatory models of innate immune cells, such as macrophages, suggest that metabolites are not merely substrates or intermediates of metabolic reactions, but may also act as signaling molecules involved in the regulation of inflammatory responses [153]. However, at present, direct causal evidence directly linking mitochondrial metabolites to NLRP3 activation in DKD remains lacking. On this basis, the following paragraph uses selected TCA cycle metabolites as examples to summarize their reported associations with DKD and integrates findings from inflammatory models of innate immune cells to discuss their potential significance as candidate metabolic nodes linking mitochondrial metabolic dysfunction to inflammasome activation.

Succinate has relatively well-established pro-inflammatory evidence in DKD, as reflected by the expression of pro-inflammatory cytokines such as TNF-α and IL-6 [156]. Through its receptor, succinate may also participate in pathological processes including RAAS activation, lipid accumulation, oxidative stress, and fibrosis [156,157]. In macrophages, succinate can also promote IL-1β expression by stabilizing HIF-1α, thereby amplifying inflammatory output [109]. In contrast, certain metabolites exert anti-inflammatory effects. Itaconate and its cell-permeable derivative, 4-octyl itaconate (4-OI), can inhibit inflammasome activation by modifying key cysteine residues in NLRP3 [111]. In a chronic renal fibrosis model, Ke et al. found that exogenous 4-OI could mimic the anti-inflammatory effects of itaconate, suppress NLRP3 inflammasome activation, and alleviate renal dysfunction and fibrosis. The same study also suggested that the inhibitory effect of dapagliflozin on the NLRP3 inflammasome is at least partly dependent on the itaconate pathway [158]. Notably, although fumarate has been reported to block pyroptosis by inhibiting GSDMD pore formation, it may also induce HIF-1α and upregulate TGF-β1 in DKD-related contexts, indicating that its biological effects are highly context dependent [110,159]. Future studies should further clarify the roles of TCA cycle–related metabolites in DKD.

### 4.5. Potential Role of the NLRP3 Inflammasome in Amplifying Mitochondrial Damage

Mitochondrial dysfunction is considered one of the major upstream triggers of NLRP3 inflammasome activation [5]. However, whether activation of the NLRP3 inflammasome can, in turn, aggravate mitochondrial damage and thereby establish a positive feedback loop of “mitochondrial damage–inflammatory activation–further mitochondrial damage” remains insufficiently supported by direct evidence in the context of DKD. Current understanding is largely derived from mechanistic studies conducted in non-DKD models. Therefore, in the setting of DKD, the NLRP3 inflammasome may contribute to the sustained amplification of mitochondrial damage; however, whether this process constitutes a complete positive feedback loop requires further support from DKD-specific evidence.

Mechanistically, downstream effector molecules of the inflammasome have the capacity to directly or indirectly disrupt mitochondrial homeostasis. Studies have shown that, during pyroptosis, cleaved GSDMD-NT can bind to cardiolipin and form pores in both the inner and outer mitochondrial membranes, thereby directly inducing mitochondrial damage. This process leads to increased ROS production, loss of mitochondrial membrane potential, impaired OXPHOS, and the release of mitochondrial proteins and mtDNA [122]. In addition, a study suggested that following NLRP3 inflammasome activation, caspase-1 not only mediates the maturation and release of inflammatory cytokines and pyroptotic cell death but may also amplify mitochondrial injury through multiple pathways. Some of these mechanisms may involve cleavage of Parkin, which weakens the clearance of damaged mitochondria and promotes the accumulation of mitochondrial injury [123].

In the field of kidney research, several studies have provided outcome-level evidence supporting this possibility. For example, Kim et al. found that *Nlrp3* deficiency enhanced mitophagy and alleviated tissue injury in a model of renal tubular epithelial cell damage associated with hypoxia/unilateral ureteral obstruction [142]. A recent study also reported that *Nlrp3* knockout was associated with attenuation of mitochondrial dynamics disturbance in a model of renal tubular epithelial cell injury [149]. These findings suggest that NLRP3 may participate in the sustained amplification of mitochondrial homeostatic imbalance in renal cells and provide indirect evidence for a bidirectional coupling relationship in DKD. Nevertheless, the existing evidence is mainly derived from non-DKD models of renal injury and remains largely limited to functional outcomes, with direct validation of the complete causal chain still lacking. Future studies should further elucidate this process in DKD-specific models by integrating different renal cell types with pathway-specific interventions.

## 5. Treatment

Increasing recognition of the mitochondria–NLRP3 inflammasome axis as a key driver of DKD progression has opened new avenues for therapeutic intervention. Here, we summarize representative drugs and bioactive molecules that have shown beneficial effects in DKD by targeting the mitochondria–NLRP3 inflammasome axis (Table 3).

### 5.1. Direct Targeting of the NLRP3 Inflammasome

In recent years, small-molecule compounds that directly inhibit the NLRP3 inflammasome have emerged as a major focus of drug development. The representative inhibitor MCC950 targets the Walker B motif in the NACHT domain of NLRP3, thereby blocking ATP hydrolysis and inhibiting NLRP3 oligomerization, inflammasome assembly, and subsequent caspase-1 activation [167]. As a result, MCC950 exhibits significant anti-inflammatory effects across multiple models of inflammatory and metabolic diseases [160]. In several mouse models of DKD, MCC950 inhibited the NLRP3/caspase-1/IL-1β axis and alleviated glomerular inflammation and fibrosis [36,161]. Moreover, in other models of kidney disease, MCC950 also attenuated tubulointerstitial inflammation and fibrosis, suggesting that, in addition to its glomerular protective effects, it may also confer potential benefits at the tubulointerstitial level in DKD [168]. Notably, reports of hepatotoxicity-related signals emerged during the early clinical development of MCC950, which, to some extent, limited its further clinical advancement and highlighted the need for careful monitoring of hepatic safety in subsequent compounds of the same class [169]. In addition to MCC950, CY-09 can specifically inhibit NLRP3 by directly binding to the ATP-binding motif within the NACHT domain of NLRP3 and suppressing its ATPase activity. In high glucose-induced models, these effects were associated with reduced renal oxidative stress, inflammation, and fibrotic progression, thereby conferring protection against DKD-related renal injury [162]. Dapansutrile (OLT1177), an orally active selective inhibitor of the NLRP3 inflammasome, has completed early-phase clinical trials in conditions such as heart failure and gout, demonstrating favorable safety and anti-inflammatory activity [170]. Dapansutrile is currently being evaluated in a clinical study (NCT06047262) targeting type 2 diabetes and its complications, and the results may provide indirect support for future clinical assessments using DKD-specific endpoints. Overall, although several direct NLRP3 inhibitors have accumulated substantial preclinical evidence in the field of DKD, their long-term safety, risk of infection, and potential effects on host defense still require careful evaluation.

### 5.2. Blockade of Downstream Effector Molecules

IL-1β and IL-18 are key downstream effector molecules activated by the NLRP3 inflammasome; therefore, targeting the IL-1/IL-18 axis holds certain translational promise. Among currently available therapeutic strategies, the IL-1 receptor antagonist anakinra, the anti-IL-1β monoclonal antibody canakinumab, and the IL-1 trap rilonacept have already been approved or clinically applied in diseases such as rheumatoid arthritis, CAPS, and gout. By contrast, IL-18-targeted inhibitory strategies remain at the clinical investigation stage. For example, the recombinant human IL-18 binding protein tadekinig alfa is currently being evaluated mainly for its therapeutic potential in IL-18-driven autoinflammatory diseases and Still’s disease [171,172,173,174]. Evidence from experimental studies indicates that anakinra markedly decreases albuminuria and mitigates mesangial matrix expansion in mouse models of DKD, with no marked effects on body weight or blood glucose in some models, suggesting that its renoprotective effects may be, at least in part, independent of metabolic improvement [23]. However, in the context of DKD, high-quality clinical evidence with renal outcomes as the primary endpoint is still lacking for interventions targeting IL-1β or IL-18. In previous large-scale clinical studies, renal function and albuminuria were mostly analyzed as secondary endpoints or exploratory measures, which remains insufficient to provide direct and robust supporting evidence [175]. In addition to targeting these cytokines, caspase-1 and the pyroptotic executor molecule GSDMD have also emerged as potential therapeutic targets [163,176]. Rat studies of DKD have indicated that VX-765, a highly selective inhibitor of caspase-1, can attenuate NOX1/ROS/NF-κB-related oxidative stress and inflammatory responses, highlighting its potential for renal protection [177]. Overall, the therapeutic potential of the aforementioned strategies in DKD has become increasingly evident. However, their efficacy and safety still need to be further confirmed by high-quality randomized controlled trials using renal outcomes as the primary endpoints.

### 5.3. Targeting Mitochondrial Function

Given the pivotal role of mitochondria in regulating NLRP3 activation, targeting mitochondrial function could serve as a potential strategy to inhibit NLRP3 and may additionally create new opportunities for multitarget intervention in DKD. While most of these approaches are still in preclinical development or early clinical evaluation, their mechanistic modulation of the mitochondria–NLRP3 inflammasome axis may better facilitate a balance between efficacy and safety.

With regard to mitochondria-targeted antioxidant strategies, MitoTEMPO effectively scavenges mtROS, suppresses renal NLRP3 inflammasome activation and the subsequent maturation and release of IL-1β, and concomitantly attenuates albuminuria and mesangial matrix expansion, without significantly altering body weight or blood glucose levels. These findings indicate that its renoprotective effects are partly attributable to inhibition of the mtROS–NLRP3–IL-1β inflammatory axis [23,48]. The mitochondria-targeted protective peptide elamipretide (SS-31) was also found to suppress NLRP3 inflammasome activation [178]. Its effects are related not only to the attenuation of oxidative stress, but also to selective binding to cardiolipin, stabilization of inner mitochondrial membrane integrity, and improvement of mitochondrial bioenergetics, thereby suggesting potential advantages in mitochondrial protection [179].

Apart from mitochondria-targeted antioxidants, schisandrin A has been shown in both in vivo and in vitro DKD models to activate the AdipoR1/AMPK signaling pathway, reduce ROS production, and alleviate mitochondrial damage, thereby mitigating pyroptosis associated with TXNIP/NLRP3 activation [164]. Similarly, astragaloside IV ameliorates glomerular podocyte injury and improves renal function in diabetic mice by modulating the TXNIP–NLRP3–GSDMD axis [131]. More recently, TIX100, a more selective orally active TXNIP inhibitor, has advanced into early clinical development, with current investigation focused primarily on type 1 diabetes mellitus, while also holding translational potential for TXNIP-targeted intervention in DKD [180].

Regarding mtDNA repair, when mtDNA undergoes oxidative damage, mitochondria primarily rely on the base excision repair (BER) pathway to recognize and repair damaged bases [136]. Therefore, in the context in which the accumulation and cytosolic translocation of ox-mtDNA contribute to NLRP3 inflammasome activation, targeting mitochondrial BER to repair oxidative mtDNA damage may help attenuate this process. 8-Oxoguanine DNA glycosylase-1 (OGG1) is a crucial DNA glycosylase in the BER pathway that recognizes and excises 8-oxoG lesions from damaged DNA, thereby initiating subsequent repair. Xian et al. reported that mtOGG1 suppressed caspase-1 activation and the processing of pro-IL-1β in bone marrow-derived macrophages by facilitating the repair of oxidized mitochondrial DNA. In addition, mitochondria-targeted *Ogg1* transgenic mice (*mt-Ogg1Tg*) likewise exhibited attenuated inflammatory responses in an alum-induced peritonitis model that was dependent on NLRP3 inflammasome activation [91]. Moreover, enhanced mitochondrial OGG1 expression has also shown anti-inflammatory effects in several other disease models [181]. Although studies in this area remain limited, the available evidence suggests that targeting the mitochondrial BER/OGG1 axis may become an important direction for DKD intervention research.

In addition to directly ameliorating mitochondrial injury, another important strategy is to enhance mitochondrial quality control. AMPK activators and SIRT1/3 agonists have been shown in multiple models to promote mitochondrial biogenesis, inhibit excessive mitochondrial fission, and enhance mitophagy, thereby reducing mtROS accumulation and mtDNA release associated with NLRP3 activation and suppressing downstream amplification of this cascade [120]. Honokiol upregulates SIRT3, preserves mitochondrial structure and membrane potential, and reduces mtROS accumulation, thereby inhibiting NLRP3 activation and subsequent GSDMD-mediated pyroptosis, ultimately ameliorating renal dysfunction and histopathological injury in diabetic mice [165]. Melatonin can activate AMPK to upregulate PINK1/Parkin-mediated mitophagy, thereby alleviating renal inflammation and interstitial fibrosis; this effect can be partially reversed by *Pink1* knockdown or AMPK inhibition [166]. The widely used antidiabetic drug metformin can also stimulate mitophagy via the AMPK–PINK1–Parkin pathway and suppress aberrant upregulation of NLRP3-related inflammatory signaling, thereby attenuating renal oxidative stress and tubulointerstitial fibrosis in HFD/STZ-induced diabetic mice [146].

## 6. Summary and Perspectives

The mitochondria–NLRP3 inflammasome axis links metabolic stress, mitochondrial injury, and inflammatory amplification, representing an important mechanism for understanding the onset and progression of DKD. However, several issues in this field remain to be clarified. First, the core mechanisms have not been fully elucidated, including the precise manner in which mitochondria serve as a platform for NLRP3 activation in the context of DKD, the regulatory effects of metabolic reprogramming and related metabolites on NLRP3, and the signaling pathways through which activated NLRP3, in turn, induces mitochondrial damage. Second, the role of this axis may differ across distinct renal cell types and stages of disease progression, and its cell-specific and stage-specific effects require further investigation.

At the same time, this axis should not be regarded as the sole mechanism underlying inflammatory injury in DKD. NLRP3 activation is also influenced by disturbances in ion homeostasis, lysosomal damage, endoplasmic reticulum stress, and other factors. Similarly, mitochondrial dysfunction may be jointly driven by lipid metabolic disorders, oxidative stress, microcirculatory impairment, and related pathological processes. Therefore, future studies should examine this axis within the broader context of the metabolic–inflammatory–microenvironmental network.

At present, the available evidence is derived mainly from animal models and in vitro experiments. Species differences, model limitations, and the complexity of human DKD pathology all constrain its clinical translation. Future basic research may integrate single-cell omics, spatial omics, multi-omics analyses, and high-resolution imaging techniques to delineate the dynamic changes in the mitochondria–NLRP3 inflammasome axis across different disease stages and nephron cell populations, thereby identifying key pathogenic nodes and cell subpopulations. In clinical studies, biomarkers related to inflammation and mitochondrial function should be incorporated, and patients should be stratified according to high-inflammatory phenotypes, mitochondrial injury phenotypes, and other relevant features, in order to improve the detectability of therapeutic signals and the interpretability of study outcomes.

Overall, the mitochondria–NLRP3 inflammasome axis provides an important perspective for understanding the inflammatory mechanisms and metabolic injury involved in DKD. Its clinical translation will require continued efforts in mechanistic elucidation, target validation, drug development, and clinical trial design.

## Figures and Tables

**Table 1:** Cell-Type Differences Related to NLRP3 in DKD.

Cell Types	Major Pathological Outputs	Refs.
Podocytes	Podocyte loss; foot process effacement; slit diaphragm disruption; proteinuria; glomerulosclerosis Impaired autophagy (via inflammasome-independent NLRP3)	[23,34,35,36,37,38]
GECs	Endothelial barrier disruption; elevated adhesion molecules; proteinuria; glomerular injury	[23,26,27,39,40]
MCs	Mesangial expansion; ECM deposition; glomerulosclerosis	[29,41,42,43,44,45]
TECs	Tubulointerstitial inflammation; oxidative stress and mitochondrial metabolic dysfunction; renal interstitial fibrosis	[46,47,48,49,50,51]
Macrophages	Paracrine crosstalk with resident renal cells; inflammatory amplification; profibrotic microenvironment formation	[52,53,54]

**Table 2:** Mitochondrial Regulation of the NLRP3 Inflammasome.

Category	Regulator	Effect on NLRP3	Principal Mechanism	Refs.
Mitochondrial danger signals	mtROS	Promotes activation	Induces TXNIP dissociation and binding to NLRP3; promotes mtDNA oxidation and cytosolic release; may also directly oxidize NLRP3	[88,89,90]
	mtDNA	Promotes activation	Cytosolic mtDNA released through mPTP/VDAC interacts with NLRP3; extracellular mtDNA, after uptake or endosomal entry, can also enhance inflammatory signaling via TLR9-related pathways, thereby promoting activation	[88,91,92,93,94,95,96,97]
Mitochondrial dynamics	DRP1-mediated mitochondrial fission	Promotes activation	Leads to excessive mitochondrial fragmentation, aggravates mitochondrial membrane potential loss, increases mtROS levels, and enhances the likelihood of mitochondrial components such as mtDNA escaping into the cytosol, thereby promoting activation	[98,99,100,101]
	MFN1/2- or OPA1-mediated mitochondrial fusion	Inhibits activation	Maintains mitochondrial fusion status, cristae structural integrity, and mitochondrial homeostasis, thereby reducing the release of mitochondrial danger signals and suppressing NLRP3 inflammasome activation	[102,103,104]
Mitophagy	PINK1/Parkin-, BNIP3-, or FUNDC1-mediated mitophagy	Inhibits activation	Clears damaged mitochondria, reduces mtROS accumulation, and limits the release of DAMPs such as mtDNA, thereby restraining sustained NLRP3 inflammasome activation at its source	[105,106,107,108]
TCA cycle intermediates	Succinate	Generally promotes activation	Stabilizes HIF-1α and induces IL-1β expression, thereby amplifying inflammatory responses	[109]
	Fumarate	May inhibit activation	Modifies GSDMD at Cys191/Cys192, thereby inhibiting pore formation and pyroptosis and attenuating downstream NLRP3 signaling output	[110]
	Itaconate and its derivative (4-OI)	Generally inhibits activation	Covalently modifies NLRP3 at Cys548, disrupts NEK7 binding, and blocks inflammasome assembly and activation	[111]
FAO-related enzymes	CPT1A	Inhibits activation	May preserve mitochondrial homeostasis through CPT1A-dependent FAO, thereby limiting mitochondria-derived danger signals, such as mtROS and ox-mtDNA	[112]
	CPT1A	Promotes activation	May promote NLRP3 inflammasome activation and assembly through FAO-dependent mitochondrial/metabolic remodeling, potentially involving ROS generation and α-tubulin acetylation	[113,114]
	HADHA	May inhibit activation	Maintains active FAO flux to preserve mitochondrial fitness and integrity, sustain membrane potential, and prevent mitochondrial fission/fragmentation and excessive mtROS generation	[115]
Mitochondria-associated platform signals	STAT3	Promotes activation	Interacts with NLRP3 and facilitates its mitochondrial localization	[116]
	MAVS	Promotes activation	Acts as a mitochondria-associated adaptor that interacts with NLRP3 and facilitates its recruitment to mitochondria	[86]
	MFN2	Promotes activation	Associates with NLRP3 and enhances NLRP3–MAVS interaction; potentially promotes NLRP3 inflammasome assembly and activation by facilitating MAMs formation	[117,118]
	Cardiolipin	Promotes activation	Redistributes to the cytosolic side of the outer mitochondrial membrane under stress conditions, thereby promoting the recruitment and binding of NLRP3 to damaged mitochondria	[119]

**Table 3:** Therapeutic interventions targeting the mitochondria–NLRP3 inflammasome axis in DKD.

Intervention	Mechanism of Action	Experimental Model(s)	Effects	Refs.
MCC950	Binds the Walker B motif within the NACHT domain of NLRP3 and inhibits ASC oligomerization	db/db mice; rat mesangial cell line (HBZY-1)	Improved renal function (serum creatinine, ACR); attenuated podocyte injury, tubular injury, renal fibrosis, and histopathological alterations; suppressed NLRP3 signaling	[160,161]
CY-09	Binds the ATP-binding motif in the NACHT domain of NLRP3 and inhibits ATPase activation	db/db mice; HK-2 cells	Improved renal function (BUN); reduced oxidative stress and alleviated tubular injury, renal fibrosis, and histopathological alterations; suppressed NLRP3 signaling and pyroptosis	[162]
VX-765	Directly targets and inhibits caspase-1, blocking inflammasome-dependent maturation and release of IL-1β/IL-18	STZ+HFD-induced diabetic Sprague–Dawley (SD) rats; HBZY-1 cells	Improved renal function (serum creatinine, UACR) and lowered serum triglyceride levels; attenuated oxidative stress and ECM accumulation; downregulated NOX1/ROS/NF-κB pathway activity	[163]
Anakinra	Competitively binds IL-1 receptor type I (IL-1RI), blocking IL-1α/IL-1β receptor binding and downstream signaling	db/db mice; unilateral nephrectomy + STZ-induced diabetic C57BL/6 mice	Reduced albuminuria and glomerular extracellular matrix accumulation	[23]
MitoTempo	Mitochondria-targeted scavenger of superoxide anions	db/db mice; unilateral nephrectomy + STZ-induced diabetic C57BL/6 mice; HK-2 cells	Reduced albuminuria and glomerular extracellular matrix accumulation; suppressed NLRP3 signaling	[23,48]
Schisandrin A	Activates the AdipoR1/AMPK pathway, thereby reducing ROS and alleviating mitochondrial damage	HFD + STZ-induced DKD mice; HRGECs	Ameliorated renal function (serum creatinine, BUN, urinary protein) and blood glucose; alleviated oxidative stress and ferroptosis; suppressed TXNIP/NLRP3 signaling and pyroptosis	[164]
Astragaloside IV	Downregulates TXNIP expression	db/db mice; conditionally immortalized mouse podocytes	Ameliorated renal function and related biochemical indices (serum creatinine, BUN, UACR, blood glucose, triglycerides); reduced oxidative stress; alleviated renal histopathological injury and fibrosis; suppressed NLRP3 signaling and pyroptosis	[46,131]
Freeze-dried powder of *Poecilobdella manillensis*	Downregulates TXNIP expression	HBZY-1 cells	Significantly inhibited expression of NLRP3, IL-1β, IL-18, and TNF-α	[45]
Tangshen formula	Downregulates TXNIP expression	Unilateral nephrectomy + STZ-induced diabetic SD rats; HK-2 cells	Attenuated oxidative stress, renal histopathological injury, and renal fibrosis; suppressed NLRP3 signaling and pyroptosis	[47]
Honokiol	Upregulates SIRT3 to maintain mitochondrial homeostasis	db/db mice; HK-2 cells	Ameliorated renal function indices (serum creatinine, BUN, 24-h urinary protein); alleviated renal histopathological injury; suppressed NLRP3 signaling and pyroptosis	[165]
Melatonin	Enhances mitophagy via the AMPK–PINK1–Parkin pathway	HFD+STZ-induced DKD mice; HK-2 cells	Ameliorated renal function and related biochemical indices (serum creatinine, BUN, 24-h urinary protein, blood glucose); reduced oxidative stress; alleviated renal histopathological injury and fibrosis; suppressed NLRP3 signaling	[166]
Icariin (ICA)	Upregulates Sesn2 to enhance mitophagy	STZ-induced diabetic SD rats; MPC-5 podocytes	Ameliorated renal function and related biochemical indices (serum creatinine, creatinine clearance, BUN, 24-h urinary protein, blood glucose, fasting insulin); attenuated renal histopathological injury; markedly reduced NLRP3–caspase-1–IL-1β pathway activity	[147]

## Data Availability

No new data were created or analyzed in this study. Data sharing is not applicable to this article.

## Abbreviations

Abbreviation	Full name
4-OI	4-octyl itaconate
AGEs	advanced glycation end products
AMPK	AMP-activated protein kinase
ASC	apoptosis-associated speck-like protein containing a CARD
ATP	adenosine triphosphate
BER	base excision repair
CX3CL1	C-X3-C motif chemokine ligand 1
DAMPs	damage-associated molecular patterns
DKD	diabetic kidney disease
DRP1	dynamin-related protein 1
dTGN	dispersed trans-Golgi network
ECM	extracellular matrix
ETC	electron transport chain
FAO	fatty acid oxidation
FEN1	flap endonuclease 1
GECs	glomerular endothelial cells
GSDMD	gasdermin D
GSDMD-NT	N-terminal fragment of gasdermin D
HIF-1α	hypoxia-inducible factor-1α
HMGB1	high mobility group box 1
IL-18	interleukin-18
IL-1R	interleukin-1 receptor
IL-1β	interleukin-1β
IL-6	interleukin-6
LC3	microtubule-associated protein 1 light chain 3
MAMs	mitochondria-associated endoplasmic reticulum membranes
MAVS	mitochondrial antiviral-signaling protein
MCP-1	monocyte chemoattractant protein 1
MCs	mesangial cells
MFN1	mitofusin 1
MFN2	mitofusin 2
mPTP	mitochondrial permeability transition pore
mTOR	mechanistic target of rapamycin
mtROS	mitochondrial reactive oxygen species
MyD88	myeloid differentiation primary response 88
NEK7	NIMA-related kinase 7
NF-κB	nuclear factor-κB
NLRP3	NOD-like receptor family pyrin domain-containing 3
Nox4	NADPH oxidase 4
Nrf2	nuclear factor erythroid 2-related factor 2
OGG1	8-oxoguanine DNA glycosylase 1
OPA1	optic atrophy 1
ox-mtDNA	oxidized mitochondrial DNA
OXPHOS	oxidative phosphorylation
PAMPs	pathogen-associated molecular patterns
PGC-1α	peroxisome proliferator-activated receptor γ coactivator-1α
PI3K	phosphatidylinositol 3-kinase
PINK1	PTEN-induced putative kinase 1
PTECs	proximal tubular epithelial cells
PTMs	post-translational modifications
PYD	pyrin domain
RAGE	receptor for advanced glycation end products
RAS	renin-angiotensin system
ROS	reactive oxygen species
Sirt1	sirtuin 1
SIRT3	sirtuin 3
SOD1	superoxide dismutase 1
SOD2	superoxide dismutase 2
SREBP1	sterol regulatory element-binding protein 1
STAT3	signal transducer and activator of transcription 3
TCA	tricarboxylic acid
TECs	tubular epithelial cells
TGF-β	transforming growth factor-β
TGN	trans-Golgi network
TLR	toll-like receptor
TNF-α	tumor necrosis factor-α
TNFR	tumor necrosis factor receptor
TRIF	TIR-domain-containing adaptor-inducing interferon-β
TRX	thioredoxin
TXNIP	thioredoxin-interacting protein
VDAC	voltage-dependent anion channel

## References

[1] Agarwal R., Filippatos G., Pitt B., Anker S.D., Rossing P., Joseph A., Kolkhof P., Nowack C., Gebel M., Ruilope L.M. (2022). Cardiovascular and kidney outcomes with finerenone in patients with type 2 diabetes and chronic kidney disease: The FIDELITY pooled analysis. Eur. Heart J..

[2] Baigent C., Emberson J., Haynes R., Herrington W.G., Judge P., Landray M.J., Mayne K.J., Ng S.Y.A., Preiss D., Roddick A.J. (2022). Impact of diabetes on the effects of sodium glucose co-transporter-2 inhibitors on kidney outcomes: Collaborative meta-analysis of large placebo-controlled trials. Lancet.

[3] Speeckaert R., Delrue C., Speeckaert M.M. (2026). Residual renal risk in diabetic nephropathy despite contemporary therapies. J. Clin. Med..

[4] Tang S.C.W., Yiu W.H. (2020). Innate immunity in diabetic kidney disease. Nat. Rev. Nephrol..

[5] Zhao W., Zhou L., Novák P., Shi X., Lin C.B., Zhu X., Yin K. (2022). Metabolic Dysfunction in the Regulation of the NLRP3 Inflammasome Activation: A Potential Target for Diabetic Nephropathy. J. Diabetes Res..

[6] Marques E., Kramer R., Ryan D.G. (2024). Multifaceted mitochondria in innate immunity. npj Metab. Health Dis..

[7] Mellacheruvu M., Lawrence G.M.E.P., Emming S., Schroder K. (2023). Reversing the mitochondrial hex that bewitches NLRP3. Sci. Immunol..

[8] Ahmad A.A., Draves S.O., Rosca M. (2021). Mitochondria in diabetic kidney disease. Cells.

[9] Ma Q. (2023). Pharmacological Inhibition of the NLRP3 Inflammasome: Structure, Molecular Activation, and Inhibitor-NLRP3 Interaction. Pharmacol. Rev..

[10] Fu J., Wu H. (2023). Structural mechanisms of NLRP3 inflammasome assembly and activation. Annu. Rev. Immunol..

[11] Wan J., Liu D., Pan S., Zhou S., Liu Z. (2022). NLRP3-mediated pyroptosis in diabetic nephropathy. Front. Pharmacol..

[12] Huang Y., Xu W., Zhou R. (2021). NLRP3 inflammasome activation and cell death. Cell. Mol. Immunol..

[13] Nie L., Fei C., Fan Y., Dang F., Zhao Z., Zhu T., Wu X., Dai T., Balasubramanian A., Pan J. (2024). Consecutive palmitoylation and phosphorylation orchestrates NLRP3 membrane trafficking and inflammasome activation. Mol. Cell.

[14] Yu T., Hou D., Zhao J., Lu X., Greentree W.K., Zhao Q., Yang M., Conde D.-G., Linder M.E., Lin H. (2024). NLRP3 Cys126 palmitoylation by ZDHHC7 promotes inflammasome activation. Cell Rep..

[15] Park Y., Dodantenna N., Kim Y., Kim T., Lee H., Yoo Y., Heo J., Lee J., Kwon M., Kang H.C. (2023). MARCH5-dependent NLRP3 ubiquitination is required for mitochondrial NLRP3-NEK7 complex formation and NLRP3 inflammasome activation. EMBO J..

[16] Dufies O., Zanoni I. (2025). Post-translational modifications of NLRP3: To prime or not to prime?. Trends Immunol..

[17] Paik S., Kim J.K., Shin H.J., Park E.-J., Kim I.S., Jo E.-K. (2025). Updated insights into the molecular networks for NLRP3 inflammasome activation. Cell. Mol. Immunol..

[18] Que X., Zheng S., Song Q., Pei H., Zhang P. (2024). Fantastic voyage: The journey of NLRP3 inflammasome activation. Genes Dis..

[19] Tschopp J., Schroder K. (2010). NLRP3 inflammasome activation: The convergence of multiple signalling pathways on ROS production?. Nat. Rev. Immunol..

[20] Paik S., Kim J.K., Silwal P., Sasakawa C., Jo E.-K. (2021). An update on the regulatory mechanisms of NLRP3 inflammasome activation. Cell. Mol. Immunol..

[21] Yang S., Huang G., Ting J. (2025). Mitochondria and NLRP3: To die or inflame. Immunity.

[22] Chen Y., Chen R., Ji X., Zeng Z., Guan C. (2025). NLRP3 inflammasome-mediated pyroptosis in diabetic nephropathy: Pathogenic mechanisms and therapeutic targets. J. Inflamm. Res..

[23] Shahzad K., Bock F., Dong W., Wang H., Kopf S., Kohli S., Al-Dabet M.M., Ranjan S., Wolter J., Wacker C. (2015). Nlrp3-inflammasome activation in non-myeloid-derived cells aggravates diabetic nephropathy. Kidney Int..

[24] Ding H., Li J., Li Y., Yang M., Nie S., Zhou M., Zhou Z., Yang X., Liu Y., Hou F.F. (2021). MicroRNA-10 negatively regulates inflammation in diabetic kidney via targeting activation of the NLRP3 inflammasome. Mol. Ther..

[25] Hou Y., Lin S., Qiu J., Sun W., Dong M., Xiang Y., Wang L., Du P. (2020). NLRP3 inflammasome negatively regulates podocyte autophagy in diabetic nephropathy. Biochem. Biophys. Res. Commun..

[26] Chen Z., Wang Z., Hu Y., Lin H., Yin L., Kong J., Zhang Y., Hu B., Li T., Zheng X. (2023). ELABELA/APJ axis prevents diabetic glomerular endothelial injury by regulating AMPK/NLRP3 pathway. Inflammation.

[27] Shao Y., Deng S., Tang W., Huang L., Xie Y., Yuan S., Tang L. (2023). Molecular mechanism of GSDMD mediated glomerular endothelial cells pyroptosis: An implying in the progression of diabetic nephropathy. Int. Immunopharmacol..

[28] Fang B., Huang W., Du S., Hao Y., He F., Zhang C. (2025). The inflammatory cell death in diabetic kidney disease: Integrating multifactorial mechanisms into novel therapeutics. Int. J. Mol. Sci..

[29] Zhan J.-F., Huang H.-W., Huang C., Hu L.-L., Xu W.-W. (2020). Long non-coding RNA NEAT1 regulates pyroptosis in diabetic nephropathy via mediating the miR-34c/NLRP3 axis. Kidney Blood Press. Res..

[30] Wang Y., Zhu X., Yuan S., Wen S., Liu X., Wang C., Qu Z., Li J., Liu H., Sun L. (2019). TLR4/NF-κB signaling induces GSDMD-related pyroptosis in tubular cells in diabetic kidney disease. Front. Endocrinol..

[31] Chen K., Zhang J., Zhang W., Zhang J., Yang J., Li K., He Y. (2013). ATP-P2X4 signaling mediates NLRP3 inflammasome activation: A novel pathway of diabetic nephropathy. Int. J. Biochem. Cell Biol..

[32] Cui X., Li Y., Yuan S., Huang Y., Chen X., Han Y., Liu Z., Li Z., Xiao Y., Wang Y. (2023). Alpha-kinase1 promotes tubular injury and interstitial inflammation in diabetic nephropathy by canonical pyroptosis pathway. Biol. Res..

[33] Cliff C.L., Squires P.E., Hills C.E. (2024). Tonabersat suppresses priming/activation of the NOD-like receptor protein-3 (NLRP3) inflammasome and decreases renal tubular epithelial-to-macrophage crosstalk in a model of diabetic kidney disease. Cell Commun. Signal. CCS.

[34] Shahzad K., Fatima S., Khawaja H., Elwakiel A., Gadi I., Ambreen S., Zimmermann S., Mertens P.R., Biemann R., Isermann B. (2022). Podocyte-specific Nlrp3 inflammasome activation promotes diabetic kidney disease. Kidney Int..

[35] Cao Y., Hu L., Chen R., Chen Y., Liu H., Wei J. (2025). Unfolded protein response-activated NLRP3 inflammasome contributes to pyroptotic and apoptotic podocyte injury in diabetic kidney disease via the CHOP-TXNIP axis. Cell. Signal..

[36] Wu M., Yang Z., Zhang C., Shi Y., Han W., Song S., Mu L., Du C., Shi Y. (2021). Inhibition of NLRP3 inflammasome ameliorates podocyte damage by suppressing lipid accumulation in diabetic nephropathy. Metabolism..

[37] Wang M.-Z., Wang J., Cao D.-W., Tu Y., Liu B.-H., Yuan C.-C., Li H., Fang Q.-J., Chen J.-X., Fu Y. (2022). Fucoidan alleviates renal fibrosis in diabetic kidney disease via inhibition of NLRP3 inflammasome-mediated podocyte pyroptosis. Front. Pharmacol..

[38] Hu X., Wang J., Jiang L., Liu X., Ge Q., Wang Q., Qi X., Wu Y. (2024). Rutaecarpine protects podocytes in diabetic kidney disease by targeting VEGFR2/NLRP3-mediated pyroptosis. Int. Immunopharmacol..

[39] Gupta A., Singh K., Fatima S., Ambreen S., Zimmermann S., Younis R., Krishnan S., Rana R., Gadi I., Schwab C. (2022). Neutrophil extracellular traps promote NLRP3 inflammasome activation and glomerular endothelial dysfunction in diabetic kidney disease. Nutrients.

[40] Wu Q., Guan Y., Zhang K., Li L., Zhou Y. (2023). Tanshinone IIA mediates protection from diabetes kidney disease by inhibiting oxidative stress induced pyroptosis. J. Ethnopharmacol..

[41] Feng H., Gu J., Gou F., Huang W., Gao C., Chen G., Long Y., Zhou X., Yang M., Liu S. (2016). High glucose and lipopolysaccharide prime NLRP3 inflammasome via ROS/TXNIP pathway in mesangial cells. J. Diabetes Res..

[42] Liu Y., Li X., Zhao M., Wu Y., Xu Y., Li X., Fu L., Han L., Zhou W., Hu Q. (2023). Macrophage-derived exosomes promote activation of NLRP3 inflammasome and autophagy deficiency of mesangial cells in diabetic nephropathy. Life Sci..

[43] Chen F., Wei G., Xu J., Ma X., Wang Q. (2018). Naringin ameliorates the high glucose-induced rat mesangial cell inflammatory reaction by modulating the NLRP3 inflammasome. BMC Complement. Altern. Med..

[44] Sun Z., Ma Y., Chen F., Wang S., Chen B., Shi J. (2018). Artesunate ameliorates high glucose-induced rat glomerular mesangial cell injury by suppressing the TLR4/NF-κB/NLRP3 inflammasome pathway. Chem. Biol. Interact..

[45] Sun X., Mijiti M., Huang C., Mei S., Fang K., Yang Y. (2024). The effect and mechanism of freeze-dried powder of poecilobdella manillensis on improving inflammatory injury of rat glomerular mesangial cells through TXNIP/NLRP3 pathway. Heliyon.

[46] Li X., Dong X., Zhang L., Zhang S., Huang W., Wang C., Huo Z., Li X., Zhang X., Jia X. (2024). Astragaloside IV attenuates renal tubule injury in DKD rats via suppression of CD36-mediated NLRP3 inflammasome activation. Front. Pharmacol..

[47] Li N., Zhao T., Cao Y., Zhang H., Peng L., Wang Y., Zhou X., Wang Q., Li J., Yan M. (2021). Tangshen formula attenuates diabetic kidney injury by imparting anti-pyroptotic effects via the TXNIP-NLRP3-GSDMD axis. Front. Pharmacol..

[48] Hou Y., Wang Q., Han B., Chen Y., Qiao X., Wang L. (2021). CD36 promotes NLRP3 inflammasome activation via the mtROS pathway in renal tubular epithelial cells of diabetic kidneys. Cell Death Dis..

[49] Wang X., Wan J., Wang C., Tong Y., Chen Y., Wang X., Liu J., Li Q., Dong Z., Hong Q. (2025). Trem2+ macrophages alleviate renal tubule lipid accumulation and ferroptosis in diabetic nephropathy by repressing IL-1β–mediated CD36 expression. Diabetes.

[50] Kong X., Zhao Y., Wang X., Yu Y., Meng Y., Yan G., Yu M., Jiang L., Song W., Wang B. (2023). Loganin reduces diabetic kidney injury by inhibiting the activation of NLRP3 inflammasome-mediated pyroptosis. Chem. Biol. Interact..

[51] Liu Y., He M., Xiong H., Yuan F. (2022). Induction of pyroptosis in renal tubular epithelial cells using high glucose. Front. Med..

[52] Kim S.-M., Lee S.-H., Kim Y.-G., Kim S.-Y., Seo J.-W., Choi Y.-W., Kim D.-J., Jeong K.-H., Lee T.-W., Ihm C.-G. (2015). Hyperuricemia-induced NLRP3 activation of macrophages contributes to the progression of diabetic nephropathy. Am. J. Physiol. Ren. Physiol..

[53] Ma Q., Xu M., Jing X., Qiu J., Huang S., Yan H., Yin L., Lou J., Zhao L., Fan Y. (2023). Honokiol suppresses the aberrant interactions between renal resident macrophages and tubular epithelial cells in lupus nephritis through the NLRP3/IL-33/ST2 axis. Cell Death Dis..

[54] Islamuddin M., Qin X. (2024). Renal macrophages and NLRP3 inflammasomes in kidney diseases and therapeutics. Cell Death Discov..

[55] Oda K., Miyamoto S., Kodera R., Wada J., Shikata K. (2022). Suramin prevents the development of diabetic kidney disease by inhibiting NLRP3 inflammasome activation in KK-ay mice. J. Diabetes Investig..

[56] Deng T., Tang C., Zhang G., Wan X. (2021). DAMPs released by pyroptotic cells as major contributors and therapeutic targets for CAR-T-related toxicities. Cell Death Dis..

[57] Cheng Q., Pan J., Zhou Z., Yin F., Xie H., Chen P., Li J., Zheng P., Zhou L., Zhang W. (2021). Caspase-11/4 and gasdermin D-mediated pyroptosis contributes to podocyte injury in mouse diabetic nephropathy. Acta Pharmacol. Sin..

[58] Meliambro K., He J.C., Campbell K.N. (2024). Podocyte-targeted therapies—Progress and future directions. Nat. Rev. Nephrol..

[59] Lin J., Cheng A., Cheng K., Deng Q., Zhang S., Lan Z., Wang W., Chen J. (2020). New insights into the mechanisms of pyroptosis and implications for diabetic kidney disease. Int. J. Mol. Sci..

[60] Novick D. (2024). IL-18 and IL-18BP: A unique dyad in health and disease. Int. J. Mol. Sci..

[61] Pereira M., Gazzinelli R.T. (2023). Regulation of innate immune signaling by IRAK proteins. Front. Immunol..

[62] Yaribeygi H., Atkin S.L., Sahebkar A. (2019). Interleukin-18 and diabetic nephropathy: A review. J. Cell. Physiol..

[63] Rayego-Mateos S., Rodrigues-Diez R.R., Fernandez-Fernandez B., Mora-Fernández C., Marchant V., Donate-Correa J., Navarro-González J.F., Ortiz A., Ruiz-Ortega M. (2023). Targeting inflammation to treat diabetic kidney disease: The road to 2030. Kidney Int..

[64] Kong H., Zhao H., Chen T., Song Y., Cui Y. (2022). Targeted P2X7/NLRP3 signaling pathway against inflammation, apoptosis, and pyroptosis of retinal endothelial cells in diabetic retinopathy. Cell Death Dis..

[65] Tang D., Kang R., Zeh H.J., Lotze M.T. (2023). The multifunctional protein HMGB1: 50 years of discovery. Nat. Rev. Immunol..

[66] Zhou T., Fang Y.-L., Tian T.-T., Wang G.-X. (2024). Pathological mechanism of immune disorders in diabetic kidney disease and intervention strategies. World J. Diabetes.

[67] Ansari Z., Chaurasia A., Neha N., Sharma N., Bachheti R.K., Gupta P.C. (2025). Exploring inflammatory and fibrotic mechanisms driving diabetic nephropathy progression. Cytokine Growth Factor Rev..

[68] Zhang H., Wang Z. (2020). Effect and regulation of the NLRP3 inflammasome during renal fibrosis. Front. Cell Dev. Biol..

[69] Vesey D.A., Cheung C., Cuttle L., Endre Z., Gobe G., Johnson D.W. (2002). Interleukin-1β stimulates human renal fibroblast proliferation and matrix protein production by means of a transforming growth factor-β-dependent mechanism. J. Lab. Clin. Med..

[70] Fan J.M., Huang X.R., Ng Y.Y., Nikolic-Paterson D.J., Mu W., Atkins R.C., Lan H.Y. (2001). Interleukin-1 induces tubular epithelial-myofibroblast transdifferentiation through a transforming growth factor-β1-dependent mechanism in vitro. Am. J. Kidney Dis..

[71] Matsui F., Rhee A., Hile K.L., Zhang H., Meldrum K.K. (2013). IL-18 induces profibrotic renal tubular cell injury via STAT3 activation. Am. J. Physiol. Ren. Physiol..

[72] Wu M., Han W., Song S., Du Y., Liu C., Chen N., Wu H., Shi Y., Duan H. (2018). NLRP3 deficiency ameliorates renal inflammation and fibrosis in diabetic mice. Mol. Cell. Endocrinol..

[73] Wang W., Wang X., Chun J., Vilaysane A., Clark S., French G., Bracey N.A., Trpkov K., Bonni S., Duff H.J. (2013). Inflammasome-independent NLRP3 augments TGF-β signaling in kidney epithelium. J. Immunol..

[74] Ding Y., Fu X., Wang Q., Liu H., Wang H., Wu D. (2021). The complex interplay between autophagy and NLRP3 inflammasome in renal diseases. Int. J. Mol. Sci..

[75] Bonam S.R., Mastrippolito D., Georgel P., Muller S. (2024). Pharmacological targets at the lysosomal autophagy–NLRP3 inflammasome crossroads. Trends Pharmacol. Sci..

[76] Han Y.-P., Liu L.-J., Yan J.-L., Chen M.-Y., Meng X.-F., Zhou X.-R., Qian L.-B. (2023). Autophagy and its therapeutic potential in diabetic nephropathy. Front. Endocrinol..

[77] Shi C.-S., Shenderov K., Huang N.-N., Kabat J., Abu-Asab M., Fitzgerald K.A., Sher A., Kehrl J.H. (2012). Activation of autophagy by inflammatory signals limits IL-1β production by targeting ubiquitinated inflammasomes for destruction. Nat. Immunol..

[78] Dong W., Jia C., Li J., Zhou Y., Luo Y., Liu J., Zhao Z., Zhang J., Lin S., Chen Y. (2022). Fisetin attenuates diabetic nephropathy-induced podocyte injury by inhibiting NLRP3 inflammasome. Front. Pharmacol..

[79] Jounai N., Kobiyama K., Shiina M., Ogata K., Ishii K.J., Takeshita F. (2011). NLRP4 negatively regulates autophagic processes through an association with Beclin1. J. Immunol..

[80] Chen W., Zhang H. (2024). Elucidating the mechanism of IL-1β-mediated Piezo1 expression regulation of chondrocyte autophagy and apoptosis via the PI3K/AKT/mTOR signaling pathway. Tissue Cell.

[81] Yu W., Haoyu Y., Ling Z., Xing H., Pengfei X., Anzhu W., Lili Z., Linhua Z. (2025). Targeting lipid metabolic reprogramming to alleviate diabetic kidney disease: Molecular insights and therapeutic strategies. Front. Immunol..

[82] Liang J.J., Fraser I.D.C., Bryant C.E. (2021). Lipid regulation of NLRP3 inflammasome activity through organelle stress. Trends Immunol..

[83] Wani K., AlHarthi H., Alghamdi A., Sabico S., Al-Daghri N.M. (2021). Role of NLRP3 Inflammasome Activation in Obesity-Mediated Metabolic Disorders. Int. J. Environ. Res. Public. Health.

[84] León-Román J., López-Martínez M., Esteves A., Ciudin A., Núñez-Delgado S., Álvarez T., Lecube A., Rico-Fontalvo J., Soler M.J. (2025). Obesity-Related Kidney Disease: A Growing Threat to Renal Health. Int. J. Mol. Sci..

[85] Yang K., Fang Y., He J., Li J. (2026). Adipokine networks in diabetic kidney disease: Mechanistic insights and therapeutic implications. Lipids Health Dis..

[86] Akbal A., Dernst A., Lovotti M., Mangan M.S.J., McManus R.M., Latz E. (2022). How location and cellular signaling combine to activate the NLRP3 inflammasome. Cell. Mol. Immunol..

[87] Gupta S., Cassel S.L., Sutterwala F.S., Dagvadorj J. (2025). Regulation of the NLRP3 inflammasome by autophagy and mitophagy. Immunol. Rev..

[88] Qiu Y., Huang Y., Chen M., Yang Y., Li X., Zhang W. (2022). Mitochondrial DNA in NLRP3 inflammasome activation. Int. Immunopharmacol..

[89] Yabal M., Calleja D.J., Simpson D.S., Lawlor K.E. (2019). Stressing out the mitochondria: Mechanistic insights into NLRP3 inflammasome activation. J. Leukoc. Biol..

[90] Cescato M., Zhu Y.Y.J., Le Corre L., Py B.F., Georgin-Lavialle S., Rodero M.P. (2024). Implication of the LRR domain in the regulation and activation of the NLRP3 inflammasome. Cells.

[91] Xian H., Watari K., Sanchez-Lopez E., Offenberger J., Onyuru J., Sampath H., Ying W., Hoffman H.M., Shadel G.S., Karin M. (2022). FEN1-generated oxidized DNA fragments exit mitochondria via mPTP- and VDAC-dependent channels to activate NLRP3 inflammasome and interferon signaling. Immunity.

[92] Baik S.H., Ramanujan V.K., Becker C., Fett S., Underhill D.M., Wolf A.J. (2023). Hexokinase dissociation from mitochondria promotes oligomerization of VDAC which facilitates NLRP3 inflammasome assembly and activation. Sci. Immunol..

[93] Nakahira K., Haspel J.A., Rathinam V.A., Lee S.-J., Dolinay T., Lam H.C., Englert J.A., Rabinovitch M., Cernadas M., Kim H.P. (2011). Autophagy proteins regulate innate immune response by inhibiting NALP3 inflammasome-mediated mitochondrial DNA release. Nat. Immunol..

[94] Cabral A., Cabral J.E., Wang A., Zhang Y., Liang H., Nikbakht D., Corona L., Hoffman H.M., McNulty R. (2023). Differential binding of NLRP3 to non-oxidized and ox-mtDNA mediates NLRP3 inflammasome activation. Commun. Biol..

[95] Zhong Z., Liang S., Sanchez-Lopez E., He F., Shalapour S., Lin X., Wong J., Ding S., Seki E., Schnabl B. (2018). New mitochondrial DNA synthesis enables NLRP3 inflammasome activation. Nature.

[96] Ward G.A., Dalton R.P., Meyer B.S., McLemore A.F., Aldrich A.L., Lam N.B., Onimus A.H., Vincelette N.D., Trinh T.L., Chen X. (2023). Oxidized mitochondrial DNA engages TLR9 to activate the NLRP3 inflammasome in myelodysplastic syndromes. Int. J. Mol. Sci..

[97] Wu G., Zhu Q., Zeng J., Gu X., Miao Y., Xu W., Lv T., Song Y. (2019). Extracellular mitochondrial DNA promote NLRP3 inflammasome activation and induce acute lung injury through TLR9 and NF-κB. J. Thorac. Dis..

[98] Zhang J., Wei Q., Wu S., Wang F., Yuan T.-L., Wang J. (2024). Inhibition of Drp1-mediated mitochondrial fission improves contrast-induced acute kidney injury by targeting the mROS-TXNIP-NLRP3 inflammasome axis. Int. Immunopharmacol..

[99] Yang N.-S.-Y., Zhong W.-J., Sha H.-X., Zhang C.-Y., Jin L., Duan J.-X., Xiong J.-B., You Z.-J., Zhou Y., Guan C.-X. (2024). mtDNA-cGAS-STING axis-dependent NLRP3 inflammasome activation contributes to postoperative cognitive dysfunction induced by sevoflurane in mice. Int. J. Biol. Sci..

[100] Li C., Zhu Y., Liu W., Hayashi T., Xiang W., He S., Mizuno K., Hattori S., Fujisaki H., Ikejima T. (2023). Increased mitochondrial fission induces NLRP3/cGAS-STING mediated pro-inflammatory pathways and apoptosis in UVB-irradiated immortalized human keratinocyte HaCaT cells. Arch. Biochem. Biophys..

[101] Sbai O., Bazzani V., Tapaswi S., McHale J., Vascotto C., Perrone L. (2023). Is Drp1 a link between mitochondrial dysfunction and inflammation in alzheimer’s disease?. Front. Mol. Neurosci..

[102] Lv Q., Hong F., Sun Z., Shen H., Lu H., Jin Y., Mao L., Song L. (2025). OPA1 modulates NLRP3 inflammasome activation and microglial-mediated neuroinflammation in neonatal hypoxic-ischemic brain injury. J. Neuroimmunol..

[103] Dong J., Bobe G., Guan Y., Li G., Zuo R., Shu X., Wang Y., Sun X., Chen X., Li X. (2020). Mitochondrial membrane protein mitofusin 2 as a potential therapeutic target for treating free fatty acid–induced hepatic inflammation in dairy cows during early lactation. J. Dairy Sci..

[104] Yang Z., Liu Y., Chen X., Huang S., Li Y., Ye G., Cao X., Su W., Zhuo Y. (2023). Empagliflozin targets Mfn1 and Opa1 to attenuate microglia-mediated neuroinflammation in retinal ischemia and reperfusion injury. J. Neuroinflamm..

[105] Xu Y., Tang Y., Lu J., Zhang W., Zhu Y., Zhang S., Ma G., Jiang P., Zhang W. (2020). PINK1-mediated mitophagy protects against hepatic ischemia/reperfusion injury by restraining NLRP3 inflammasome activation. Free Radic. Biol. Med..

[106] Bu W., Li S., Liu C., Wang Y., Lu J., Dong C., Zheng D., Fan Z., Yu Y., Zhang W. (2025). Nepetin limits NLRP3 inflammasome activation and alleviates NLRP3-driven inflammatory diseases via PINK1-dependent mitophagy. Free Radic. Biol. Med..

[107] Lin Q., Li S., Jiang N., Jin H., Shao X., Zhu X., Wu J., Zhang M., Zhang Z., Shen J. (2021). Inhibiting NLRP3 inflammasome attenuates apoptosis in contrast-induced acute kidney injury through the upregulation of HIF1A and BNIP3-mediated mitophagy. Autophagy.

[108] Lin Q., Li S., Jiang N., Shao X., Zhang M., Jin H., Zhang Z., Shen J., Zhou Y., Zhou W. (2019). PINK1-parkin pathway of mitophagy protects against contrast-induced acute kidney injury via decreasing mitochondrial ROS and NLRP3 inflammasome activation. Redox Biol..

[109] Tannahill G., Curtis A., Adamik J., Palsson-McDermott E., McGettrick A., Goel G., Frezza C., Bernard N., Kelly B., Foley N. (2013). Succinate is a danger signal that induces IL-1β via HIF-1α. Nature.

[110] Humphries F., Shmuel-Galia L., Ketelut-Carneiro N., Li S., Wang B., Nemmara V.V., Wilson R., Jiang Z., Khalighinejad F., Muneeruddin K. (2020). Succination inactivates gasdermin D and blocks pyroptosis. Science.

[111] Hooftman A., Angiari S., Hester S., Corcoran S.E., Runtsch M.C., Ling C., Ruzek M.C., Slivka P.F., McGettrick A.F., Banahan K. (2020). The immunomodulatory metabolite itaconate modifies NLRP3 and inhibits inflammasome activation. Cell Metab..

[112] Liu Y., Liu Y., Yu X., Tian S., Li X., Gao Y., Bao X., Wu X., Zhang B., Huang W. (2025). CPT1A mediated preservation of mitochondrial inhibits pyroptosis in pancreatic acinar cells. Front. Cell Dev. Biol..

[113] Qiao S., Lv C., Tao Y., Miao Y., Zhu Y., Zhang W., Sun D., Yun X., Xia Y., Wei Z. (2020). Arctigenin disrupts NLRP3 inflammasome assembly in colonic macrophages via downregulating fatty acid oxidation to prevent colitis-associated cancer. Cancer Lett..

[114] Li X., Zhang X., Xia J., Zhang L., Chen B., Lian G., Yun C., Yang J., Yan Y., Wang P. (2021). Macrophage HIF-2α suppresses NLRP3 inflammasome activation and alleviates insulin resistance. Cell Rep..

[115] Chi Z., Chen S., Xu T., Zhen W., Yu W., Jiang D., Guo X., Wang Z., Zhang K., Li M. (2020). Histone deacetylase 3 couples mitochondria to drive IL-1β-dependent inflammation by configuring fatty acid oxidation. Mol. Cell.

[116] Luo L., Wang F., Xu X., Ma M., Kuang G., Zhang Y., Wang D., Li W., Zhang N., Zhao K. (2024). STAT3 promotes NLRP3 inflammasome activation by mediating NLRP3 mitochondrial translocation. Exp. Mol. Med..

[117] Liu Q., Zhang D., Hu D., Zhou X., Zhou Y. (2018). The role of mitochondria in NLRP3 inflammasome activation. Mol. Immunol..

[118] Ichinohe T., Yamazaki T., Koshiba T., Yanagi Y. (2013). Mitochondrial protein mitofusin 2 is required for NLRP3 inflammasome activation after RNA virus infection. Proc. Natl. Acad. Sci. USA.

[119] Iyer S.S., He Q., Janczy J.R., Elliott E.I., Zhong Z., Olivier A.K., Sadler J.J., Knepper-Adrian V., Han R., Qiao L. (2013). Mitochondrial cardiolipin is required for Nlrp3 inflammasome activation. Immunity.

[120] Yang Y., Liu J., Shi Q., Guo B., Jia H., Yang Y., Fu S. (2024). Roles of Mitochondrial Dysfunction in Diabetic Kidney Disease: New Perspectives from Mechanism to Therapy. Biomolecules.

[121] Takasu M., Kishi S., Nagasu H., Kidokoro K., Brooks C.R., Kashihara N. (2024). The Role of Mitochondria in Diabetic Kidney Disease and Potential Therapeutic Targets. Kidney Int. Rep..

[122] Miao R., Jiang C., Chang W.Y., Zhang H., An J., Ho F., Chen P., Zhang H., Junqueira C., Amgalan D. (2023). Gasdermin D permeabilization of mitochondrial inner and outer membranes accelerates and enhances pyroptosis. Immunity.

[123] Yu J., Nagasu H., Murakami T., Hoang H., Broderick L., Hoffman H.M., Horng T. (2014). Inflammasome activation leads to caspase-1–dependent mitochondrial damage and block of mitophagy. Proc. Natl. Acad. Sci. USA.

[124] Dikalov S.I., Nazarewicz R.R., Bikineyeva A., Hilenski L., Lassègue B., Griendling K.K., Harrison D.G., Dikalova A.E. (2014). Nox2-induced production of mitochondrial superoxide in angiotensin II-mediated endothelial oxidative stress and hypertension. Antioxid. Redox Signal..

[125] Dikalov S., Dikalova A. (2022). Mitochondrial deacetylase Sirt3 in vascular dysfunction and hypertension. Curr. Opin. Nephrol. Hypertens..

[126] Long Y., Liu X., Tan X., Jiang C., Chen S., Liang G., He X., Wu J., Chen T., Xu Y. (2020). ROS-induced NLRP3 inflammasome priming and activation mediate PCB 118- induced pyroptosis in endothelial cells. Ecotoxicol. Environ. Saf..

[127] Liu X., Zhang X., Ding Y., Zhou W., Tao L., Lu P., Wang Y., Hu R. (2017). Nuclear factor E2-related factor-2 negatively regulates NLRP3 inflammasome activity by inhibiting reactive oxygen species-induced NLRP3 priming. Antioxid. Redox Signal..

[128] Juliana C., Fernandes-Alnemri T., Kang S., Farias A., Qin F., Alnemri E.S. (2012). Non-transcriptional priming and deubiquitination regulate NLRP3 inflammasome activation. J. Biol. Chem..

[129] Zhu K., Jin X., Chi Z., Chen S., Wu S., Sloan R.D., Lin X., Neculai D., Wang D., Hu H. (2021). Priming of NLRP3 inflammasome activation by msn kinase MINK1 in macrophages. Cell. Mol. Immunol..

[130] An X., Zhang Y., Cao Y., Chen J., Qin H., Yang L. (2020). Punicalagin protects diabetic nephropathy by inhibiting pyroptosis based on TXNIP/NLRP3 pathway. Nutrients.

[131] Hu Z., Zhou Y., Gao C., Liu J., Pan C., Guo J. (2024). Astragaloside IV attenuates podocyte apoptosis via regulating TXNIP/NLRP3/GSDMD signaling pathway in diabetic nephropathy. Diabetol. Metab. Syndr..

[132] Liu Z., Cheng P., Feng T., Xie Z., Yang M., Chen Z., Hu S., Han D., Chen W. (2023). Nrf2/HO-1 blocks TXNIP/NLRP3 interaction via elimination of ROS in oxygen-glucose deprivation-induced neuronal necroptosis. Brain Res..

[133] Zhang Q., Hu Y., Hu J.-E., Zhang M. (2022). Solasonine alleviates high glucose-induced podocyte injury through increasing Nrf2-medicated inhibition of NLRP3 activation. Drug Dev. Res..

[134] Du L., Wang J., Chen Y., Li X., Wang L., Li Y., Jin X., Gu X., Hao M., Zhu X. (2020). Novel biphenyl diester derivative AB-38b inhibits NLRP3 inflammasome through Nrf2 activation in diabetic nephropathy. Cell Biol. Toxicol..

[135] Dikalova A.E., Pandey A., Xiao L., Arslanbaeva L., Sidorova T., Lopez M.G., Billings F.T., Verdin E., Auwerx J., Harrison D.G. (2020). Mitochondrial deacetylase Sirt3 reduces vascular dysfunction and hypertension while Sirt3 depletion in essential hypertension is linked to vascular inflammation and oxidative stress. Circ. Res..

[136] Xian H., Karin M. (2023). Oxidized mitochondrial DNA: A protective signal gone awry. Trends Immunol..

[137] Huang L., Chen X., Shao Y., Deng S., Wang C., Chen J., Xie Y., Yuan S., Tang L. (2025). Elevation of ISG15 promotes diabetic kidney disease by modulating renal tubular epithelial cell pyroptosis. Clin. Transl. Med..

[138] Yang X., Chen Z., Luo Z., Yang D., Hao Y., Hu J., Feng J., Zhu Z., Luo Q., Zhang Z. (2023). STING deletion alleviates podocyte injury through suppressing inflammation by targeting NLRP3 in diabetic kidney disease. Cell. Signal..

[139] Zang N., Cui C., Guo X., Song J., Hu H., Yang M., Xu M., Wang L., Hou X., He Q. (2022). cGAS-STING activation contributes to podocyte injury in diabetic kidney disease. iScience.

[140] Chen K., Tang W., Liu X. (2025). Research and progress of cGAS/STING/NLRP3 signaling pathway: A mini review. Front. Immunol..

[141] Jiang X., Huang W., Wen W., Liu T., Ren W., Shi J., He J., Du X. (2026). sEH inhibition attenuates mtROS-mediated NLRP3 inflammasome activation by promoting mitophagy in tubular epithelial cells in diabetic kidney disease. Front. Immunol..

[142] Kim S.-M., Kim Y.G., Kim D.-J., Park S.H., Jeong K.-H., Lee Y.H., Lim S.J., Lee S.-H., Moon J.-Y. (2018). Inflammasome-independent role of NLRP3 mediates mitochondrial regulation in renal injury. Front. Immunol..

[143] Chun J., Chung H., Wang X., Barry R., Taheri Z.M., Platnich J.M., Ahmed S.B., Trpkov K., Hemmelgarn B., Benediktsson H. (2016). NLRP3 localizes to the tubular epithelium in human kidney and correlates with outcome in IgA nephropathy. Sci. Rep..

[144] Arumugam S., Qin Y., Liang Z., Han S.-N., Boodapati S.L.T., Li J., Lu Q., Flavell R.A., Mehal W.Z., Ouyang X. (2022). GSK3β mediates the spatiotemporal dynamics of NLRP3 inflammasome activation. Cell Death Differ..

[145] Yue R., Yan Z., Zha H., Xia Y., Huang H., Li H., Ding M., Abdullah M.M.H., Hu B., Shi L. (2025). Inhibition of Drp1-mediated mitochondrial fission by P110 ameliorates renal injury in diabetic nephropathy. Int. Immunopharmacol..

[146] Han Y., Tang S., Liu Y., Li A., Zhan M., Yang M., Song N., Zhang W., Wu X., Peng C. (2021). AMPK agonist alleviate renal tubulointerstitial fibrosis via activating mitophagy in high fat and streptozotocin induced diabetic mice. Cell Death Dis..

[147] Ding X., Zhao H., Qiao C. (2022). Icariin protects podocytes from NLRP3 activation by Sesn2-induced mitophagy through the Keap1-Nrf2/HO-1 axis in diabetic nephropathy. Phytomedicine.

[148] Kim K., Lee E.-Y. (2021). Excessively enlarged mitochondria in the kidneys of diabetic nephropathy. Antioxidants.

[149] Nam B.Y., Jhee J.H., Park J., Kim S., Kim G., Park J.T., Yoo T.-H., Kang S.-W., Yu J.-W., Han S.H. (2022). PGC-1α inhibits the NLRP3 inflammasome via preserving mitochondrial viability to protect kidney fibrosis. Cell Death Dis..

[150] Chen K., Chen J., Wang L., Yang J., Xiao F., Wang X., Yuan J., Wang L., He Y. (2020). Parkin ubiquitinates GATA4 and attenuates the GATA4/GAS1 signaling and detrimental effects on diabetic nephropathy. FASEB J..

[151] Fan X., Yang M., Lang Y., Lu S., Kong Z., Gao Y., Shen N., Zhang D., Lv Z. (2024). Mitochondrial metabolic reprogramming in diabetic kidney disease. Cell Death Dis..

[152] Choi Y.J., Richard G., Zhang G., Hodgin J.B., Demeke D.S., Yang Y., Schaub J.A., Tamayo I.M., Gurung B.K., Naik A.S. Attenuated kidney oxidative metabolism in young adults with type 1 diabetes. J. Clin. Investig. 134.

[153] Olona A., Leishman S., Anand P.K. (2022). The NLRP3 inflammasome: Regulation by metabolic signals. Trends Immunol..

[154] Wolf A.J., Reyes C.N., Liang W., Becker C., Shimada K., Wheeler M.L., Cho H.C., Popescu N.I., Coggeshall K.M., Arditi M. (2016). Hexokinase Is an Innate Immune Receptor for the Detection of Bacterial Peptidoglycan. Cell.

[155] Li H., Li D., Ledru N., Xuanyuan Q., Wu H., Asthana A., Byers L.N., Tullius S.G., Orlando G., Waikar S.S. (2024). Transcriptomic, epigenomic, and spatial metabolomic cell profiling redefines regional human kidney anatomy. Cell Metab..

[156] Wang Y., Zhang X., Yao H., Chen X., Shang L., Li P., Cui X., Zeng J. (2022). Peroxisome-generated succinate induces lipid accumulation and oxidative stress in the kidneys of diabetic mice. J. Biol. Chem..

[157] Pu M., Zhang J., Hong F., Wang Y., Zhang C., Zeng Y., Fang Z., Qi W., Yang X., Gao G. (2024). The pathogenic role of succinate-SUCNR1: A critical function that induces renal fibrosis via M2 macrophage. Cell Commun. Signal..

[158] Ke Q., Shi C., Lv Y., Wang L., Luo J., Jiang L., Yang J., Zhou Y. (2022). SGLT2 inhibitor counteracts NLRP3 inflammasome *via* tubular metabolite itaconate in fibrosis kidney. FASEB J..

[159] You Y.-H., Quach T., Saito R., Pham J., Sharma K. (2016). Metabolomics reveals a key role for fumarate in mediating the effects of NADPH oxidase 4 in diabetic kidney disease. J. Am. Soc. Nephrol..

[160] Bakhshi S., Shamsi S. (2022). MCC950 in the treatment of NLRP3-mediated inflammatory diseases: Latest evidence and therapeutic outcomes. Int. Immunopharmacol..

[161] Zhang C., Zhu X., Li L., Ma T., Shi M., Yang Y., Fan Q. (2019). A small molecule inhibitor MCC950 ameliorates kidney injury in diabetic nephropathy by inhibiting NLRP3 inflammasome activation. Diabetes Metab. Syndr. Obes. Targets Ther..

[162] Yang M., Zhao L. (2023). The selective NLRP3-inflammasome inhibitor CY-09 AmelioratesKidney injury in diabetic nephropathy by inhibiting NLRP3-inflammasome activation. Curr. Med. Chem..

[163] Luo Y., Long M., Wu X., Zeng L. (2025). Targeting the NLRP3 inflammasome in kidney disease: Molecular mechanisms, pathogenic roles, and emerging small-molecule therapeutics. Front. Immunol..

[164] Wang X., Li Q., Sui B., Xu M., Pu Z., Qiu T. (2022). Schisandrin A from Schisandra chinensis Attenuates Ferroptosis and NLRP3 Inflammasome-Mediated Pyroptosis in Diabetic Nephropathy through Mitochondrial Damage by AdipoR1 Ubiquitination. Oxid. Med. Cell. Longev..

[165] Yu K., Liu M., Zhang T., Guo C., Du L., Li M., Wang Q., Han N., Chi Y., Li Y. (2025). Honokiol attenuates diabetic nephropathy by targeting SIRT3 to suppress mitochondrial ROS-induced pyroptosis. Diabetol. Metab. Syndr..

[166] Tang H., Yang M., Liu Y., Zhu X., Liu S., Liu H., Sun L., Song P. (2022). Melatonin alleviates renal injury by activating mitophagy in diabetic nephropathy. Front. Endocrinol..

[167] Coll R.C., Hill J.R., Day C.J., Zamoshnikova A., Boucher D., Massey N.L., Chitty J.L., Fraser J.A., Jennings M.P., Robertson A.A.B. (2019). MCC950 directly targets the NLRP3 ATP-hydrolysis motif for inflammasome inhibition. Nat. Chem. Biol..

[168] You D., Nie K., Wu X., Weng M., Yang L., Chen Y., Cui J., Wan J. (2023). C3a/C3aR synergies with TGF-β to promote epithelial-mesenchymal transition of renal tubular epithelial cells via the activation of the NLRP3 inflammasome. J. Transl. Med..

[169] Cabral J.E., Wu A., Zhou H., Pham M.A., Lin S., McNulty R. (2025). Targeting the NLRP3 inflammasome for inflammatory disease therapy. Trends Pharmacol. Sci..

[170] Bai F., Wang D., Wu Y. (2025). Dapansutrile in multidisciplinary therapeutic applications: Mechanisms and clinical perspectives. Front. Pharmacol..

[171] Wang K., Luo H., Liu L., Gao H., Song Y., Li D. (2025). Blockade of IL-1 family cytokines in the treatment of rheumatoid arthritis. Front. Pharmacol..

[172] Li C., Sun M., Liu Z., Li D., Wang C., Tian Z., Dai Y., Feng Z., Xu C., Zhao D. (2025). 2024 update of chinese guidelines for management of hyperuricemia and gout part II: Recommendations for patients with common comorbidities. Int. J. Rheum. Dis..

[173] Romano M., Arici Z.S., Piskin D., Alehashemi S., Aletaha D., Barron K.S., Benseler S., Berard R., Broderick L., Dedeoglu F. (2022). The 2021 EULAR/american college of rheumatology points to consider for diagnosis, management and monitoring of the interleukin-1 mediated autoinflammatory diseases: Cryopyrin-associated periodic syndromes, tumour necrosis factor receptor-associated periodic syndrome, mevalonate kinase deficiency, and deficiency of the interleukin-1 receptor antagonist. Ann. Rheum. Dis..

[174] Landy E., Carol H., Ring A., Canna S. (2024). Biologic and clinical roles of IL-18 in inflammatory diseases. Nat. Rev. Rheumatol..

[175] Ridker P.M., MacFadyen J.G., Glynn R.J., Koenig W., Libby P., Everett B.M., Lefkowitz M., Thuren T., Cornel J.H. (2018). Inhibition of Interleukin-1β by Canakinumab and Cardiovascular Outcomes in Patients with Chronic Kidney Disease. J. Am. Coll. Cardiol..

[176] Hu J.J., Liu X., Xia S., Zhang Z., Zhang Y., Zhao J., Ruan J., Luo X., Lou X., Bai Y. (2020). FDA-approved disulfiram inhibits pyroptosis by blocking gasdermin D pore formation. Nat. Immunol..

[177] Wang X., Wu T., Ma H., Huang X., Huang K., Ye C., Zhu S. (2022). VX-765 ameliorates inflammation and extracellular matrix accumulation by inhibiting the NOX1/ROS/NF-κB pathway in diabetic nephropathy. J. Pharm. Pharmacol..

[178] Li M., Kong D., Meng L., Wang Z., Bai Z., Wu G. (2024). Discovery of novel SS-31 (d-arg-dimethylTyr-lys-phe-NH_2_) derivatives as potent agents to ameliorate inflammation and increase mitochondrial ATP synthesis. RSC Adv..

[179] Sabbah H.N., Alder N.N., Sparagna G.C., Bruce J.E., Stauffer B.L., Chao L.H., Pitceathly R.D.S., Maack C., Marcinek D.J. (2025). Contemporary insights into elamipretide’s mitochondrial mechanism of action and therapeutic effects. Biomed. Pharmacother..

[180] Jing G., Jo S., Shalev A. (2024). A novel class of oral, non-immunosuppressive, beta cell-targeting, TXNIP-inhibiting T1D drugs is emerging. Front. Endocrinol..

[181] Hussain M., Chu X., Sahbaz B.D., Gray S., Pekhale K., Park J.-H., Croteau D.L., Bohr V.A. (2023). Mitochondrial OGG1 expression reduces age-associated neuroinflammation by regulating cytosolic mitochondrial DNA. Free Radic. Biol. Med..

